# PHD1-3 oxygen sensors in vivo—lessons learned from gene deletions

**DOI:** 10.1007/s00424-024-02944-x

**Published:** 2024-03-21

**Authors:** Agnieszka E. Jucht, Carsten C. Scholz

**Affiliations:** 1 Institute of Physiology, University of Zurich, Zurich, 8057 Switzerland; 2 Institute of Physiology, University Medicine Greifswald, Friedrich-Ludwig-Jahn-Str. 15a, 17475 Greifswald, Germany

**Keywords:** Hypoxia, Egln, HIF, Hydroxylase inhibitor, Mouse, Knockout

## Abstract

Oxygen sensors enable cells to adapt to limited oxygen availability (hypoxia), affecting various cellular and tissue responses. Prolyl-4-hydroxylase domain 1–3 (PHD1-3; also called Egln1-3, HIF-P4H 1–3, HIF-PH 1–3) proteins belong to the Fe^2+^- and 2-oxoglutarate-dependent dioxygenase superfamily and utilise molecular oxygen (O_2_) alongside 2-oxoglutarate as co-substrate to hydroxylate two proline residues of α subunits of the dimeric hypoxia inducible factor (HIF) transcription factor. PHD1-3-mediated hydroxylation of HIF-α leads to its degradation and inactivation. Recently, various PHD inhibitors (PHI) have entered the clinics for treatment of renal anaemia. Pre-clinical analyses indicate that PHI treatment may also be beneficial in numerous other hypoxia-associated diseases. Nonetheless, the underlying molecular mechanisms of the observed protective effects of PHIs are only partly understood, currently hindering their translation into the clinics. Moreover, the PHI-mediated increase of Epo levels is not beneficial in all hypoxia-associated diseases and PHD-selective inhibition may be advantageous. Here, we summarise the current knowledge about the relevance and function of each of the three PHD isoforms in vivo, based on the deletion or RNA interference-mediated knockdown of each single corresponding gene in rodents. This information is crucial for our understanding of the physiological relevance and function of the PHDs as well as for elucidating their individual impact on hypoxia-associated diseases. Furthermore, this knowledge highlights which diseases may best be targeted by PHD isoform-selective inhibitors in case such pharmacologic substances become available.

## PHD1-3-mediated regulation of HIF

**Fig. 1:**
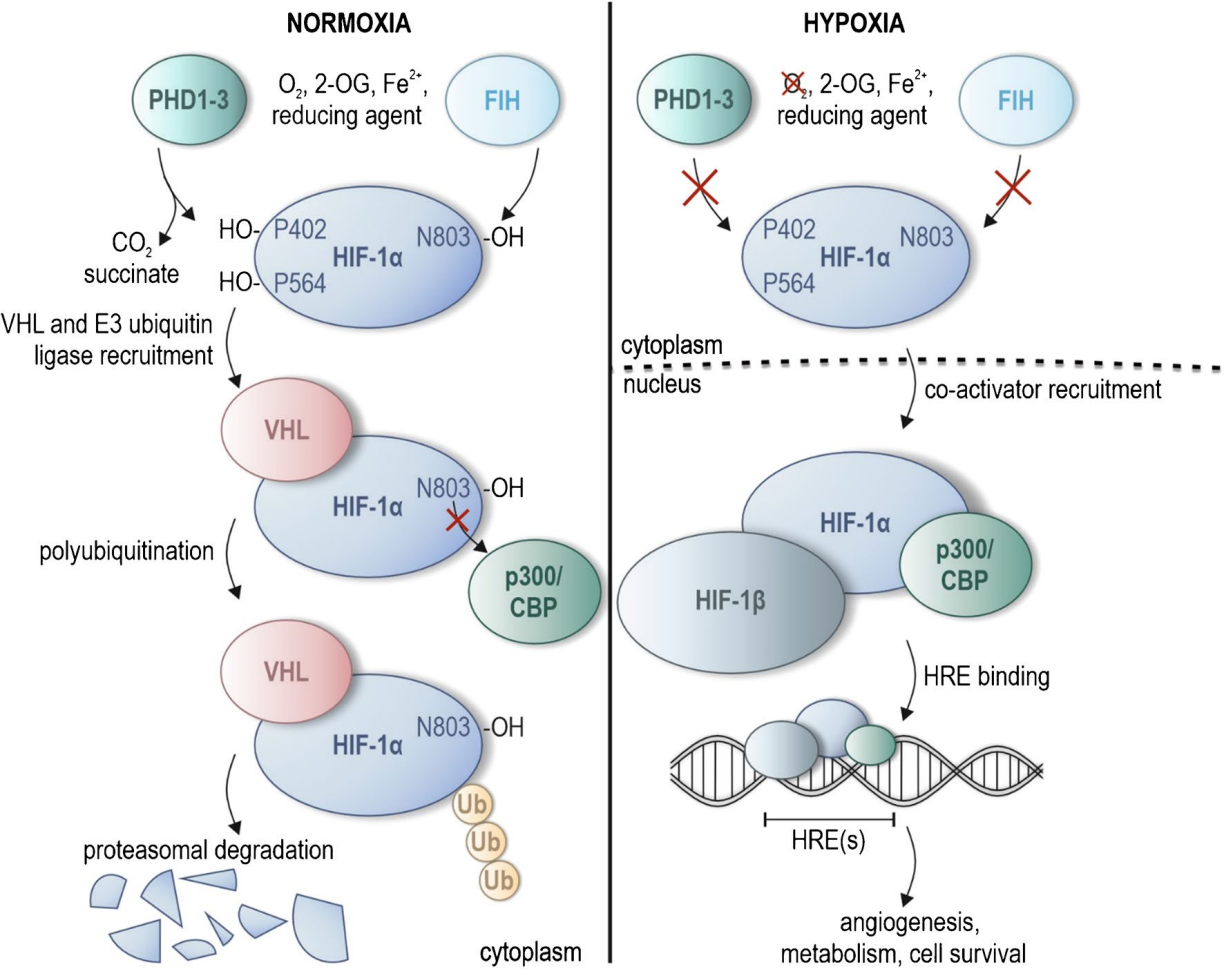
Regulation of HIF-1α by the cellular oxygen sensors PHD1-3 and FIH. Prolyl-4-hydroxylase domain (PHD) 1–3 proteins need Fe^2+^ and a reducing agent (such as ascorbate) as co-factors and use O_2_ and 2-oxogluterate (2-OG) as co-substrates. In normoxia, PHD1-3 activity leads to the hydroxylation of the proline residues Pro402 and Pro564 of hypoxia-inducible factor (HIF)-1α, leading to the binding of the von-Hippel Lindau (VHL) protein and the recruitment of an E3 ubiquitin ligase complex. Subsequently, HIF-1α is polyubiquitinated and degraded by the proteasome, preventing transactivation of genes by the HIF-1 transcription factor. FIH decreases transactivation activity of HIF-1 towards selective genes by hydroxylating asparagine N803, preventing the recruitment of the transcriptional co-activators p300/CBP to the HIF-1 transcription factor. In hypoxia, PHD1-3 and FIH can no longer catalyse the hydroxylation reaction due to the absence of O_2_, allowing HIF-1α to translocate to the nucleus, to dimerise with HIF-1β, to recruit transcriptional co-activators and to bind to selected hypoxia-response elements (HREs) to enhance gene expression

Prolyl-4-hydroxylase domain (PHD) proteins 1–3 are cellular oxygen sensors that have first been discovered to confer hypoxia sensitivity to the hypoxia-inducible factor (HIF) transcription factors [12, 36, 58–60]. The PHDs enable the tissue and cellular adaptation to hypoxia via HIF-mediated enhancement of the expression of selected genes [63, 132, 161]. There are three known α (HIF-1α, HIF-2α and HIF-3α) and one HIF-β subunit, of which one α together with the β subunit form the dimeric HIF transcription factors HIF-1, HIF-2 or HIF-3 [39, 131, 132]. HIF-1α and HIF-2α are well-characterised, but less is known about HIF-3α. The following description focusses on HIF-1α as a best described example of the PHD-dependent regulation of HIF-α subunits. In normoxia, PHD1-3 hydroxylate two proline residues of HIF-1α, Pro402 and Pro564 (Fig. 1) [63]. Prolyl hydroxylated HIF-1α is recognised by the von Hippel-Lindau protein (VHL), which in turn recruits an E3 ubiquitin ligase [47, 63, 131, 132]. HIF-1α is then polyubiquitinated and subsequently degraded by the proteasome (Fig. 1), preventing HIF-mediated enhancement of gene expression [47, 63, 131, 132].

PHD1-3 belong to the Fe^2+^ and 2-oxyglutarate (2-OG)-dependent dioxygenase superfamily [63]. The PHDs utilise molecular oxygen (O_2_) as co-substrate and thus depend on the availability of O_2_ for their enzymatic activity [63]. HIF-α subunits are also regulated by an additional cellular oxygen sensor, the asparagine hydroxylase factor inhibiting HIF (FIH, Fig. 1) [168]. FIH belongs to the same superfamily as the PHDs and utilises O_2_ for its enzymatic activity, hydroxylating the asparagine residue Asn803 of HIF-1α [88, 132, 168]. HIF-1α asparagine hydroxylation abrogates binding of the histone acetyl transferases CBP/p300 that serve as transcriptional co-activators, therefore decreasing HIF-1 activity towards selected genes [88, 132, 168]. In hypoxia, the enzymatic activity of the PHDs and FIH is reduced; HIF-1α is therefore stabilised and migrates into the nucleus, forming the active HIF-1 heterodimer with HIF-1β (Fig. 1) [63]. HIF-1 then binds to hypoxia response elements (HREs), enhancing the transcription of hundreds of genes involved in various processes, including angiogenesis and energy metabolism [63, 132, 161].

Interestingly, the three PHD isoforms have shown different preferences towards the two HIF-1α prolyl hydroxylation sites. PHD1 and PHD2 hydroxylate both Pro402 and Pro564 of HIF-1α, whilst PHD3 preferentially modifies Pro564 [13, 50]. In addition, PHD2 and PHD3 gene expression is enhanced by HIF-1, forming a negative feedback loop for the regulation of HIF activity, whereas PHD1 gene expression is not altered in hypoxia [98, 142]. Of note, PHD2 is almost ubiquitously expressed, whereas the expression of PHD1 and PHD3 is more restricted [87].

Currently, six different PHD inhibitors (PHIs) are available in the clinics for treatment of renal anaemia: roxadustat, molidustat, vadadustat, daprodustat, desidustat and enarodustat [74]. PHI treatment increases the expression of the HIF-2 target gene erythropoietin (Epo) and therefore the amount of circulating red blood cells, counteracting renal anaemia [37]. Some selectivity of these drugs towards specific PHDs has been reported [101]. Roxadustat and enarodustat have been suggested to inhibit all PHDs to a comparable degree, whereas daprodustat preferentially inhibits PHD1 and 3, and molidustat shows a preference towards PHD2 and vadadustat for PHD3 [101]. However, such analyses are based on the investigation of purified enzymes and whether this apparent PHI selectivity also occurs in vivo is currently unclear, as it depends on the expression level of the corresponding PHDs together with the reached concentration of the PHIs in the targeted cells. Of note, all of these inhibitors increase the expression of Epo in the human kidney (which is quintessential for their use as treatment of renal anaemia) and PHD2 is the most relevant PHD for the regulation of Epo expression [25, 48]. Therefore, all currently available PHIs in the clinics must inhibit PHD2 within human renal Epo-producing cells in vivo. A potential differential selectivity may lead to diverse side effects, but the currently available data do not allow such conclusions and more studies are necessary.

Alongside the well-characterised regulation of HIF-α by the PHDs, it has also been reported that the PHDs regulate proteins outside the HIF pathway [80, 143]. A PHD-mediated regulation of substrates other than HIF has obviously profound implications for our understanding of the cellular adaptation to hypoxia as well as for the use of pharmacologic PHD inhibitors. However, the hydroxylation of non-HIF-α proteins by the PHDs is currently controversially discussed [7, 22, 80, 143], as it was not possible to reproduce these findings with purified proteins in vitro [22]. The assessment of the phenotype(s) of PHD knockout (KO) animals will contribute to solving this discussion, as the regulation of a target protein by one of the PHDs should ultimately be linked to the in vivo﻿ function of the respective PHD.

The function of each of the PHDs in vivo is of major relevance for our understanding of the regulation of the tissue and cellular response to hypoxia and to elucidate potential novel treatment options for hypoxia-associated diseases as well as for the understanding of possible side effects. In the following, we therefore summarise to the best of our knowledge the currently described phenotypes in rodents with deletion or RNA interference-mediated knockdown of single PHD isoforms. Regarding the relevance of PHD1-3 in cancer, different outcomes have been reported, depending on whether a PHD-encoding gene was deleted in tumour cells, in the host organism or in both [75]. The function of the PHDs and the HIF pathway in cancer has recently been expertly reviewed elsewhere [43, 106, 172]. In the chapters about cancer in this review, only reports are summarised that describe the effects of *Phd* gene inactivation in the host organism.

## *Phd1 (Egln2)* deletion

The baseline phenotype summarises observations made in mice with *Phd1* deletion without the induction of a pathology. The subsequent chapters focus on phenotypes of mice with various *Phd1* deletions in disease models.

### Baseline phenotype

Analysis of mice with constitutive global deletion of single *Phd* genes gives key insights into the functional role and relevance of the respective protein and can indicate what (side) effects may occur following treatment with a (currently not available) PHD isoform-selective pharmacologic inhibitor. Mice with constitutive whole-body deficiency of *Phd1* (*Phd1*^−/−^) do not display any obvious phenotype under normal housing conditions during development [152] or adulthood [1, 4, 97, 149, 150], with intact skeletal muscles [4] and erythropoiesis (haematocrit, haemoglobin and erythropoietin levels) as well as normal blood gas values [4, 103], vascular system [150], heart [1], lung [117] and liver [91, 107, 130] morphology. Also, bone development and architecture were unaltered as shown in *Phd1*^−/−^ mice and by conditional deletion of *Phd1* in osteoprogenitors (OSX-Cre) or chondrocytes (Col2α1-Cre) [55, 173, 178]. Interestingly, in *Phd1*^−/−^ mice whole-body oxygen consumption was reduced at rest and the mice demonstrated worse exercise endurance and impaired oxidative muscle performance due to a decreased oxygen consumption in skeletal muscle [4]. During aging, 1-year-old *Phd1*^−/−^ mice showed a decreased serum cholesterol level and a reduced body weight [158]. The size and frequency of pulmonary neuroepithelial bodies (NEB; presumed hypoxia-sensitive oxygen sensors) were increased in *Phd1*^−/−^ mice [116, 117] combined with an enhanced NEB serotonin (5-HT) production in normoxia and hypoxia [93]. NEBs may have functional relevance for the ventilatory response to hypoxia, especially perinatally [24]; nonetheless, no difference has been reported in the viability of *Phd1*^−/−^ mice. In addition, the hypoxia ventilatory response in adult *Phd1*^−/−^ mice is not altered [9].

In contrast to other observations, one group reported that *Phd1*^−/−^ mice exhibit a lower body weight, food intake and liver weight [162], albeit the liver over body weight ratio was not altered [107]. Despite the decreased body weight, adult *Phd1*^−/−^ mice displayed a larger white adipose tissue (WAT) mass, altered glucose homeostasis and a decreased insulin sensitivity [162]. Interestingly, β-cell-specific *Phd1* deletion (Ins1-Cre) led to decreased β-cell mass and elevated β-cell apoptosis, but not to any defect in glucose homeostasis or insulin sensitivity [51].

In summary, *Phd1* deletion does not affect the majority of organs at baseline, but impacts on energy metabolism, which however does not appear to be obvious without challenge.

### Cardiovascular system

HIF activity is tightly linked to angiogenesis through regulation of the expression of corresponding genes, including vascular endothelial growth factor (VEGF) [185]. In addition, in tissue ischemia (as it, e.g. occurs during a so-called heart attack), the resulting hypoxia affects the activity of the PHDs and leads to stabilisation of HIF-α. This led to the analyses of the relevance of the different PHD proteins in ischemic diseases including the heart.

*Phd1*^−*/*−^ mice display a reduced infarct size after ischemia/reperfusion (I/R) injury (Langendorff’s perfusion model) with decreased apoptosis in cardiomyocytes [1]. DNA-binding activity of HIF-1α is increased in *Phd1*^−*/*−^ hearts following I/R, which was associated with enhanced expression of anti-apoptotic BCL-2 and endothelial nitric oxide synthase (eNOS). In addition, the DNA-binding activity of NF-κB was augmented as well as the nuclear translocation of β-catenin in cardiac tissue [1]. Based on this study, PHD1 may be a relevant pharmaceutical target in myocardial I/R injury. Interestingly, using whole-body inducible shRNA targeting both PHD1 and PHD3 in combination in mice, it was found that knockdown of PHD1 and PHD3 was not protective against myocardial infarction [61]. It remains unclear if the protective effect of *Phd1* deletion alone is time-dependent and does not occur in acute settings or if the combinatorial deletion of both *Phd1* and *Phd3* has a different effect than *Phd1* ablation alone. Of note, *Phd3* KO alone has also been reported to be protective in cardiac ischemia (see below).

Combined deletion of *Phd1* and low-density lipoprotein receptor (*Ldlr*) in mice led to decreased artherosclerotic plague sizes and reduced plasma cholesterol levels compared to *Ldlr*^−*/*−^ mice, which was likely caused by enhanced cholesterol excretion into the intestines [97]. HIF-2α silencing via antisense oligonucleotides had no effect on the protection conferred by *Phd1* deletion, indicating a HIF-2α-independent mechanism [97]. Conditional deletion of *Phd1* in myeloid cells (LysM-Cre) had no effect on aortic plaque size or plaque type following a high-cholesterol diet [166]. In summary, whole-body *Phd1* KO is advantageous in atherosclerosis, which is likely due to an altered regulation of plasma cholesterol levels.

### Haematopoietic system

Constitutive whole-body [149], hepatic (Alb-Cre) [103, 163] or FOXD1 lineage cell (Foxd1-Cre) [73] deletion of *Phd1* did not affect Epo or haematocrit levels.

### Liver

Livers from *Phd1*^−/−^ mice are largely protected against acute ischemia and I/R injury [130]. Hypoxic cell damage following ischemia, including hepatocyte swelling, vascularisation and karyolysis, was markedly decreased in the livers from *Phd1*^−/−^ mice [130], suggesting a higher tolerance to hypoxia. In addition, mice lacking *Phd1* were protected against hepatocyte apoptosis and necrosis, with reduced oxidative stress following I/R and decreased oxygen consumption [130]. Interestingly, both at baseline conditions and following I/R, global *Phd1* KO led to higher HIF-2α than HIF-1α protein levels [130].

Simultaneous silencing (via tail vein injection of shRNAs) of *Phd1* and *Keap1* (an oxidative stress sensor) in hepatocytes reduced hepatic fibrosis induced by treatment with carbon tetrachloride (CCl_4_) [91]. Liver fibrosis was also attenuated in *Phd1*^−/−^ mice following the induction of chronic bile duct injury through application of 3,5-diethoxycarbonyl-1,4-dihydrocollidine (DDC) [145]. Livers of *Phd1*^−/−^ mice displayed a reduced recruitment of inflammatory leukocytes as well as a decreased number of profibrotic myofibroblasts combined with a lower expression of pro-mitogenic and pro-fibrogenic factors. These effects were likely caused (at least in part) by a mitigated activation of hepatic stellate cells [145].

After 80% hepatectomy in mice, *Phd1*^−/−^ animals recovered their liver weight significantly faster than WT through increased proliferation of hepatocytes [107]. Enhanced proliferation in *Phd1*^−/−^ hepatocytes was caused by increased HIF-2 and c-Myc activity [107]. Following low-fat diet (LFD), *Phd1*^−/−^ mice developed hepatic steatosis with increased hepatic cholesterol and triglyceride (TG) content [162].

In summary, PHD1 deletion is protective in various liver pathologies, ranging from I/R injury over fibrosis to hepatectomy. The observation that *Phd1* deletion may lead to hepatic steatosis may have to be taken into account, when PHD1 is considered therapeutic target. However, all phenotypes based on *Phd1* deletion that could be used to reason against PHD1 as therapeutic target have been reported in a single publication, whereas the majority of investigations observed a protective effect of *Phd1* KO in liver pathologies.

### Energy metabolism

Following LFD, *Phd1*^−*/*−^ mice demonstrated an increased body weight gain combined with a decreased insulin sensitivity [162]. Following high-fat diet (HFD), *Phd1*^−/−^ mice displayed a larger body weight gain, but decreased cholesterol and blood glucose levels as well as an improved insulin sensitivity compared to wildtype (WT) mice [162]. Another study also reported that *Phd1*^−/−^ mice are protected against HFD-induced glucose intolerance and hyperglycaemia [97]. Overall, the effect of PHD1 on energy metabolism may be diet-dependent, but PHD1 is generally linked to the regulation of body weight gain, glucose homeostasis and insulin sensitivity and deletion of *Phd1* (and therefore potentially also PHD1 inhibition) is protective under conditions of high-fat diet.

### Immune system

In *Phd1*^−/−^ mice, no difference was found in their response to LPS-induced sepsis relative to wildtype mice [69]. *Phd1* KO in haematopoietic and endothelial cells (Tie2-Cre) favoured a polarisation of macrophages in response to LPS towards a M2 phenotype with reduced secretion of chemokines [167]. Dendritic cells with *Phd1* deletion (CD11c-Cre) demonstrated also a decreased response to LPS with reduced IL-1β secretion [167]. Overall, few studies have analysed the effect of *Phd1* deletion on immune cell function in vivo with contrasting results (in different disease and KO mouse models). Therefore, further studies would be necessary to clarify the relevance of PHD1 for the immune system in vivo.

### Nervous system

*Phd1* deficiency did not affect the outcome of transient focal cerebral ischaemia in the first 24 h after 45 min of middle cerebral artery occlusion (MCAO) [14]. Interestingly, *Phd1* deletion was protective in a model of permanent brain ischemia 24 h after MCAO, decreasing the infarct size [120]. *Phd1* KO increased the activity of the pentose phosphate pathway and enhanced the cellular redox buffering capacity. Therefore, neurons lacking *Phd1* were protected from reactive oxygen species (ROS) [120]. The protection against brain ischemia by *Phd1* KO was suggested to be dependent on NF-κB activity and independent of HIF [120]. The long non-coding RNA (lncRNA) myocardial infarction associated transcript (MIAT) is upregulated in brain tissue after ischemic stroke. Following 90 min of MCAO in rats followed by reperfusion, overexpression of MIAT promoted I/R injury by enhancing infarct volume, neuron damage and apoptosis [85]. When MIAT overexpression was combined with *Phd1* knockdown (intravenous injection), the degenerative effects of MIAT were abrogated, suggesting that *Phd1* knockdown is protective [85]. In summary, PHD1 deletion is protective in ischemic brain injury, but only during long durations of ischemia. Interestingly, following peripheral (sciatic) nerve injury, *Phd1*^*−/−*^ mice showed reduced cold hyperalgesia combined with increased axonal regeneration [139], indicating that pharmaceutical PHD1 inhibition may be a treatment option for peripheral nerve injury.

### Skeletal muscle

*Phd1*^*−*/*−*^ mice are protected against hind limb ischemia. Following femoral artery ligation, there was almost no necrosis or apoptosis detected within the skeletal muscle of *Phd1*^*−*/*−*^ mice [4]. *Phd1* deletion reduced oxidative stress and mitochondrial damage in ischemic myofibres, whereas ATP production was maintained, demonstrating a hypoxia tolerance of myofibres when *Phd1* was absent [4]. Hypoxia tolerance and fibre protection were linked to upregulation of the metabolic regulator Pparα and elevated HIF-2α levels [4]. Another study analysing the effect of femoral artery ligation in *Phd1*^*−*/*−*^ mice found increased motor function as well as improved recovery of perfusion together with increased arteriolar and capillary density, capillary/myocyte ratio and decreased fibrosis [126]. Acute decrease of PHD1 expression via shPHD1 injection (into both gastrocnemius and tibial anterior muscles) after femoral artery ligation increased leg capillary density; however, the proangiogenic effect was not as strong as with shPHD2 or shPHD3, which was likely regulated via stabilisation of HIF-1α [94]. Overall, a lack of *Phd1* is protective against ischemic injury in skeletal muscle due to an altered energy metabolism and an enhanced angiogenesis.

### Gastrointestinal tract

*Phd1*^*−/−*^ mice are protected against dextran sulphate sodium (DSS)-induced colitis [154]. *Phd1* KO increased colonic epithelial cell density and enhanced epithelial barrier function caused by decreased epithelial cell apoptosis [154]. Weight loss, disease activity index and colonic pro-inflammatory cytokines were reduced in the absence of *Phd1* [154]. Conditional deletion of *Phd1* in endothelial and haematopoietic cells was also protective against DSS-induced colitis [167]. Haematopoietic *Phd1* deletion alone but not endothelial-selective deletion was sufficient for the protective effect, which promoted an anti-inflammatory M2 macrophage polarisation [167]. In a model of radiation-induced gastrointestinal toxicity, the deletion of *Phd1* alone in the gastrointestinal epithelium (Villin-Cre) had no effect [157]. Haematopoietic *Phd1* deletion (Vav-Cre) was not protective in a genetic mouse model of ileitis, induced by chronically enhanced production of the pro-inflammatory cytokine TNFα [28]. *Phd1* deletion did also not affect ischemic colonic anastomoses but reduced the bursting pressure in septic colonic anastomoses [144]. However, disease activity or survival was not altered by *Phd1* deletion in the septic colonic anastomoses model [144]. In a colon-associated colorectal cancer model (CAC; azoxymethane (AOM) and DSS treatment), *Phd1*^*−/−*^ mice were again protected against colitis and demonstrated a reduced CAC growth [67]. In summary, these data suggest that PHD1 is a crucial regulator of colitis, affecting both the epithelial barrier as well as the inflammatory response. In addition, CAC growth was diminished, which is associated with prolonged colon inflammation. For colon anastomoses or in ileitis, PHD1 appears to not play a relevant role in disease progression.

### Skin

In a model of acute skin inflammation (2-O-tetradecanoylphorbol-13-acetate treatment), *Phd1*^*−*/*−*^ mice showed a decreased inflammatory response combined with an increased apoptosis [164], indicating PHD1 as a potential pharmaceutical target protein in skin inflammation.

### Cancer

PHD1 expression can be induced by oestrogen [3, 133]. Interestingly, global *Phd1* deletion improved survival in triple-negative breast cancer (TNBC) mice compared to wildtype [148]. Long-term but not short-term survival was improved, indicating that loss of *Phd1* might be protective against TNBC only in slower growing tumours [148]. As indicated above, constitutive global *Phd1* deletion in mice decreased CAC growth [67]. In summary, based on the few existing studies, *Phd1* deletion and hence possibly also pharmacologic inhibition of PHD1 are protective at least in breast and colon cancer.

## *Phd2* (Egln1) deletion

*Phd2* deletion leads to multiple different phenotypes depending on whether the KO occurred prior to, during or after development and depending on the cell type(s) targeted for *Phd2* ablation. The baseline phenotype chapter summarises observations that have been made in mice with constitutive homozygous whole-body deletion of *Phd2* (*Phd2*^−/−^) and in *Phd2* hypomorph mice under baseline conditions. Analyses of heterozygous (*Phd2*^+/−^) and conditional *Phd2* KO mice in normal housing conditions or any available *Phd2* deletion in mice in disease conditions are described in the subsequent chapters.

### Baseline phenotype

Global *Phd2* KO is lethal in mice during embryogenesis [104, 152]. *Phd2*^−/−^ embryos die between embryonic days 12.5 and 14.5 due to placental and heart defects. Defects of the placenta ranged from widespread penetration of the labyrinth by spongiotrophoblasts, decreased labyrinthine branching morphogenesis to abnormal trophoblast giant cell distribution [152]. Developmental heart defects included a thinner myocardium, underdeveloped trabeculae, an incompletely formed interventricular septum and an enlarged intraventricular lumen [152]. HIF-α protein levels were increased in the embryo with the exception of the heart [152]. PHD2 knockdown in one-cell murine zygotes by injection of lentiviruses carrying shPHD2 was lethal in some but not all developing embryos on embryonic day 14 [115]. The lethality was linked to placental and heart malformations similar to the observations made in mice with constitutive global *Phd2* inactivation [115]. Of note, also induced somatic deletion of *Phd2* (chicken-β-actin-CreER) is lethal in mice due to dilated cardiomyopathy and venous congestion [104].

Hypomorphic inactivation of *Phd2* does not result in embryonic lethality, polycythaemia, enhanced angiogenesis or dilated cardiomyopathy [56]. Nonetheless, HIF-1α and HIF-2α protein levels as well as the expression of glycolytic enzymes were upregulated in the heart. Interestingly, hypomorphic *Phd2* mice display no difference in their life span compared to wildtype mice, but demonstrate a reduced occurrence of liver diseases, inflammation and myocardial infarction without effect on cancer incidence [78]. During aging, hypomorphic *Phd2* mice also demonstrated an improved diastolic function (1-year-old mice) and developed less cardiomyocyte hypertrophy (2-year-old mice) [127]. This effect was likely due to increased Notch signalling and Notch target gene expression [127].

### Cardiovascular system

Induced global inactivation of *Phd2* (ROSA26-CreERT2) in adult mice increased angiogenesis and angiectasia [150]. Moreover, mice with induced somatic *Phd2* deletion (chicken β-actin-CreER, first tamoxifen application in utero) developed dilated cardiomyopathy [104]. Induction of global *Phd2* deletion (chicken β-actin-CreER) at 3 weeks of age did not lead to a change in systolic function in 10-week-old mice with an only minimally enlarged heart [105], indicating that both the timing and duration of *Phd2* deletion are relevant for the development of the phenotype. Interestingly, constitutive cardiac-specific deletion of *Phd2* (αMHC-Cre) did not lead to a cardiac phenotype in mice [108].

Knockdown of PHD2 via intraperitoneal injection of small interfering RNAs (siRNAs) into mice reduced acute myocardial I/R injury [110, 111]. The infarct size was smaller and HIF-1α levels were increased in cardiac tissue [110]. PHD2 knockdown also decreased the infiltration of polymorphonuclear leukocytes into cardiac tissue as well as chemokine and ICAM-1 expression [111]. Intraventricular infusion of siPHD2 [34] as well as intramyocardial injection of shPHD2 [54] also decreased the myocardial infarct size. In addition, following intramyocardial injection of shPHD2, fractional shortening was improved and more small capillaries and venules were present in the infarct zone several weeks after the initial injury [54]. Following the induction of increased cardiac afterload in mice with cardiac-specific deletion of *Phd2* (αMHC-Cre), the mice developed a cardiac hypertrophy and a more profound decompensation than control mice [108]. Interestingly, constitutive cardiac-specific deletion of *Phd2* using MLCv-Cre transgenic mice did not lead to a differential response to increased afterload [53]. Following acute myocardial ischemic injury, these mice were protected displaying a decreased infarct size, a reduced number of apoptotic cells and an improved cardiac function 3 weeks after ligation of the left anterior descending (LAD) artery [53]. A separate investigation further supported that cardiomyocyte-specific *Phd2* deletion was protective in the LAD ligation-mediated ischemic injury model [119].

Isolated hearts from *Phd2* hypomorph mice (92% reduction of cardiac PHD2 mRNA) were protected against induced I/R injury (induced during Langendorff’s perfusion), demonstrating a decreased infarct size, enhanced recovery of coronary flow and mechanical function [56]. Following LAD ligation, *Phd2* hypomorph mice also displayed a reduced infarct size, an improved preservation of the systolic function of the left ventricle and an increased survival [68]. The number of cardiac capillaries was not altered but their size was increased together with an enhanced expression of endothelial HIF target genes [68].

Inducible whole-body shRNA-mediated knockdown of *Phd2* in mice was protective in acute myocardial infarction [61]. This mouse model allowed the efficient induction of a *Phd2* knockdown without increasing the haematocrit, an otherwise potentially confounding factor for analyses of the heart. *Phd2* knockdown was only present during application of doxycycline and could therefore also be switched off [61]. Inhibition of PHD2 expression for 4 weeks prior and 6 weeks after acute myocardial infarction improved left ventricular ejection fraction and fractional area shortening without affecting the diastolic function [61]. Knockdown of *Phd2* for 4 weeks prior to LAD-ligation decreased the infarct size but did not affect cardiac performance. Downregulation of *Phd2* for 2 and 6 weeks after acute myocardial infarction improved left ventricular ejection fraction and fractional area shortening [61]. Overall, these results support the hypothesis that pharmacologic PHD2-selective pharmacologic inhibition is a novel treatment option in cardiac I/R injury.

HFD in mice hypomorphic for *Phd2* combined with the deletion of the LDL receptor led to 50% reduction of atherosclerotic plaque areas, increased autoantibodies against oxidised LDL and reduced macrophage numbers in white adipose tissue without effect on serum cholesterol [123]. High-cholesterol diet in mice with conditional myeloid-specific *Phd2* inactivation increased aortic root plaque size, decreased the macrophage content and enhanced fibrosis in plaques [166]. Conditional global *Phd2* KO mice (Rosa26-CreERT2) were also protected against HFD-induced cardiac dysfunction [186]. In a murine model of hypertension-induced cardiovascular remodelling and fibrosis, myeloid-specific *Phd2* deletion (LysM-Cre) was protective [57]. The mice displayed reduced cardiomyocyte hypertrophy and cardiac interstitial fibrosis combined with a decreased aortic thickening and macrophage infiltration [57]. *Phd2* deletion in myeloid cells (LysM-Cre) combined with LDL receptor deletion enhanced angiogenesis and vessel maturation and reduced intra-plague haemorrhage within plagues that were formed following vein graft surgery into the carotid artery [138]. Following the induction of thrombosis using a combination of endothelial activation and flow restriction in vivo, induced global *Phd2* KO (ROSA26-CreERT2) had no effect on venous thrombus neovascularisation, thrombus resolution or macrophage infiltration [44]. Together, these results indicate that *Phd2* deletion is protective in diet-induced atherosclerosis and cardiac dysfunction.

Pulmonary arterial hypertension (PAH) is a common cause for right-sided heart failure. Endothelial cell-specific *Phd2* KO in mice (Cdh5-Cre) resulted in spontaneous severe PAH [35, 65, 171] with premature mortality [65]. The muscularisation of pulmonary arteries was increased, the respiratory basement membrane was thickened and a right ventricular hypertrophy was developed [35, 65, 171]. In addition, alveolar fibrosis was observed [35]. The development of PAH was dependent on HIF-2α and independent of HIF-1α [65]. Using Tie2-Cre-mediated endothelial and haematopoietic cell-specific *Phd2* deletion, the development of PAH including pulmonary vascular remodelling and right ventricular hypertrophy [27, 118, 155] as well as premature mortality [27] was also observed. In addition, in this model, it was found that the development of PAH was dependent on HIF-2α [27, 155, 191], which in turn may affect bone morphogenic protein (BMP) signalling [27, 89]. In contrast to the studies mentioned above, one group reported that conditional deletion of *Phd2* in endothelial cells via Tie2-Cre leads to cardiac fibrosis and left ventricular hypertrophy [26].

Conditional inactivation of *Phd2* in smooth muscle cells (smmhc-CreERT2) aggravated established PAH and increased hypoxia-induced vascular remodelling [15]. Using a novel Angpt4-Cre transgenic mouse line to inactivate *Phd2* in arterial smooth muscle cells, an elevated right ventricular pressure was observed as well as a change in the vascular tone [35].

In summary, PHD2 is a key enzyme for the cardiovascular system with multiple effects on heart tissue and vessels. Inhibition of PHD2 is protective in acute myocardial ischemia, but the timing of PHD2 inhibition is relevant for a successful treatment. PHD2 inhibition in the heart in diseases with increased cardiac afterload is less promising. Conditional deletion of *Phd2* in pulmonary endothelial and smooth muscle cells demonstrated that PHD2 plays a key role for the regulation of pulmonary arterial pressure and leads to the development of PAH via stabilisation of HIF-2α and pulmonary arterial remodelling. Moreover, PHD2 inhibition appears to be protective against the detrimental effects of HFD on the cardiovascular system.

### Haematopoietic system

Conditional whole-body deletion of *Phd2* increases plasma Epo levels, red blood cell (RBC) count haemoglobin and haematocrit levels [86, 104, 105, 113, 149, 152]. Mice with somatic inactivation of *Phd2* died prematurely, due to dilated cardiomyopathy and venous congestion [104]. The congestive heart failure was likely caused by blood hyperviscosity and volume overload [104]. Acute global deletion of *Phd2* led to erythrocytosis in both young (6–8 months) as well as aging (16–20 months) mice [86]. Injection of an adenovirus encoding a Cre enzyme into the tail vein of *Phd2*^*flox/flox*^ mice resulted in increased serum Epo levels [121]. Epo mRNA was increased within the liver in the acute phase of *Phd2* deletion; however, this was lost over time as long as functional PHD1 and PHD3 were present [121].

In mice with conditional *Phd2* inactivation in renal EPO-producing cells, neurons and astrocytes (hCD68-IVS1-Cre), strong EPO production was observed combined with erythrocytosis [41]. Epo production in these mice was HIF-2α dependent [41]. To assess the first described human mutation in the *Phd2* gene, which was associated with erythrocytosis, a mouse model was generated with a corresponding P294R knock-in mutation in the murine *Phd2* locus (*Phd2*^*P294R/*+^ mice) [5]. *Phd2*^*P294R/*+^ mice displayed comparable erythrocytosis levels to *Phd2*^+/−^ mice, which were HIF-2α-dependent [5]. In addition, homozygous and heterozygous *Phd2* inactivation in renal cortical interstitial cells (Pax3-Cre) also induced erythrocytosis as well as homozygous *Phd2* deletion in haematopoietic progenitor cells (Vav1-Cre) [5]. Conditional *Phd2* deletion in renal FOXD1 stroma-derived cells (Foxd1-Cre) increased plasma Epo levels, haemoglobin and haematocrit [73]. HIF-2α protein levels were enhanced in renal FOXD1 cells with targeted *Phd2* deletion, whereas HIF-1α protein levels were not altered [73]. Following obstructive nephropathy in mice with FOXD1-targeted *Phd2* ablation, Epo expression was also increased but was not linked to myofibroblast transdifferentiation [71]. Interestingly, conditional deletion of *Phd2* in the liver (Alb-Cre) did not enhance Epo levels or haematocrit [32, 103, 163]. These studies demonstrated that PHD2 is an important regulator of Epo via stabilisation of HIF-2α in specific Epo-producing cells in the kidney. Of note, inducible inactivation of *Phd2* in renin-producing cells (mRen-rtTAm2 LC1-Cre) failed to induce Epo expression, whereas simultaneous deletion of both PHD2 and PHD3 increased Epo expression in juxtaglomerular and hyperplastic renin-positive cells [11], indicating that also renin-producing cells can induce the expression of Epo.

Induced global inactivation of *Phd2* also increased the number of white blood cells in peripheral blood, which was combined with a strong increase of haematopoietic progenitors and haematopoietic stem cells in the spleen as well as a moderate but significant increase in liver and bone marrow [149]. Conditional deletion of *Phd2* in early haematopoietic precursor cells (CD68-Cre) led to HIF-1α- and SMAD7-dependent self-renewal of multipotent progenitors [136]. Overall, these studies laid the foundation for our understanding about PHD2 as the key oxygen sensor for the regulation of Epo and therefore of RBC count, haemoglobin and haematocrit in vivo, which in turn affects the oxygen transport capacity of the blood. PHD2 also directly affects haematopoietic precursor cells and PHD2 is therefore a critical enzyme for the entire haematopoietic system.

### Kidney

In HFD-fed mice with tamoxifen-mediated induction of proximal tubule (PT)-specific *Phd2* deletion (N-Myc downstream-regulated gene 1 (Ndrg1)-CreER), peritubular capillary density was increased compared to HFD-fed control mice and HIF-target gene expression was enhanced [42]. Moreover, PT-specific *Phd2*-deletion reduced tubular damage, glomerulomegaly and albuminuria [42]. Therefore, PHD2 inhibition might be a treatment option for obesity-induced kidney injury.

Analysing the relevance of the PHD isoforms for kidney development, it was demonstrated that *Phd2* deletion in FOXD1-expressing renal stroma cells (Foxd1-Cre) does not affect nephrogenesis [72]. Combined deletion of both *Phd2* and *Phd3*, however, led to abnormal development of the kidney [72].

In an endothelial-specific *Phd2* KO (VE-Cadherin (Cdh5)-Cre) mouse model, increased serum creatinine was observed together with arteriolar remodelling and increased interstitial fibrosis under normal animal housing conditions [170]. This was accompanied by glomerular arteriolar remodelling and increased renal interstitial fibrosis [170]. The *Phd2* KO upregulated transforming growth factor-β (TGF-β) and Notch3 expression, which was suggested to be linked to the increased renal interstitial fibrosis [170]. In an angiotensin II (ANG II)-mediated renal injury and fibrosis model, *Phd2*-specific KO in endothelial cells (Cdh5-Cre) reduced iron accumulation and ROS formation in the kidney and was protective against renal fibrosis [189]. This was accompanied by increased HIF-1α and HIF-2α levels as well as a decreased ANGII type 1 receptor expression in endothelial cells [189], suggesting that PHD2 activity contributes to ANGII-mediated renal fibrosis and injury. Interestingly, in renal I/R injury, endothelial-specific *Phd2* ablation (Cdh5-Cre) was protective, preserving kidney function and preventing the transition from acute kidney injury (AKI) to chronic kidney disease (CKD) in a HIF-1-dependent manner [124]. Induction of acute *Phd2* deletion in endothelial cells (Cdh5(PAC)-CreERT2) indicated that the protective effect was independent of haematopoietic cells [124].

In summary, PHD2 inactivation in endothelial cells can lead to the development of interstitial fibrosis, but is protective against both ANG-II- and I/R-mediated renal injury and fibrosis. The vast majority of investigations with pharmacologic hydroxylase inhibitors have also reported a protective effect in various models of CKD [37], overall supporting that PHD2 is a pharmaceutical relevant target for the treatment of kidney injury and disease.

### Liver

PHD2 activity in the liver plays an important role in the regulation of whole-body energy metabolism as described in the corresponding section below. Both in mice with induced systemic (chicken β-actin-Cre) and constitutive liver-specific (Alb-Cre) *Phd2* deletion, mild hepatic steatosis was observed [105]. Murine *Phd2* haploinsufficiency had no effect on liver regeneration following partial hepatectomy [107] or on fibrosis development during chronic bile duct injury [145]. Endothelial *Phd2* KO in mice (Cdh5-Cre) led to liver steatosis and fibrosis combined with enhanced fat to body weight ratio and impaired glucose tolerance [190]. HFD did not lead to an additional aggravation of the steatosis [190]. In a model for alcoholic fatty liver disease (AFLD), *Phd2* hypomorph mice displayed a decrease in adiposity, a maintained insulin sensitivity, an improved lipoprotein profile and a reduced WAT inflammation in comparison to mice with *Phd2* [79]. In addition, the mice were protected against alcohol-induced liver damage and steatosis [79]. Thus, PHD2 plays an important role in the maintenance of liver tissue homeostasis and inactivation of hepatic PHD2 increases steatosis and potentially fibrosis under baseline conditions. However, PHD2 inhibition may be protective in liver diseases, such as AFLD.

### Energy metabolism

Mice with adipocyte-specific *Phd2* inactivation (aP2-Cre) displayed no obvious phenotype under standard housing condition [99]. Following HFD, the mice displayed a reduced weight gain compared to the control with decreased WAT weight and adipocyte size as well as increased blood glucose clearance [99]. Oxygen consumption was enhanced combined with a reduced respiratory exchange ratio (RER) during darkness [99]. In adipocytes, the expression of glycolytic enzymes and adiponectin was enhanced together with an increased expression of uncoupling protein-1 (Ucp-1) in brown adipose tissue (BAT) [99]. Analysis of adipocyte-specific *Phd2* deletion in mice using fatty acid binding protein 4 (Fapb4)-Cre showed an increased adiposity and adipose vascularisation, normal glucose homeostasis and reduced circulating fatty acid levels with feeding of normal chow [102]. *Phd2* ablation in BAT by *in-situ* injection of a virus containing targeted sgRNA reduced BAT thermogenesis in cold temperatures and increased HFD-mediated weight gain [83]. Endothelial *Phd2* KO in mice (Cdh5-Cre) impaired glucose tolerance and enhanced the fat to body weight ratio [190].

Hypomorph *Phd2* mice under both normal chow and HFD showed a decrease in adipocyte size, WAT weight, WAT inflammation, improved insulin sensitivity and glucose tolerance, reduced de novo lipid synthesis and decreased serum cholesterol levels [122]. Hypomorph *Phd2* mice with additional deficiency of the LDL receptor were protected against HFD, displaying a reduced weight gain, WAT and liver weight, insulin resistance, adipocyte size, serum cholesterol levels and number of macrophages within WAT [123]. Induced global *Phd2* KO (ROSA-CreERT2) in mice also decreased body weight gain and improved glucose tolerance after HFD [186]. Moreover, conditional whole-body KO of *Phd2* led to more successful endurance training and faster running time [113]. Pancreatic β-cell specific *Phd2* KO (insulin-1 promoter (Ins-1)-Cre) in mice resulted in glucose-induced increases in plasma insulin [51].

In mice with induced global *Phd2* deletion (chicken β-actin-CreER), lactate levels were decreased compared to control mice after treadmill exercise [146]. Liver-specific *Phd2* ablation (Alb-Cre) in mice led to an activation of the Cori cycle, which serves as a recycling system of lactate-glucose carbon between the muscles and liver [146]. Consequently, mice with hepatic *Phd2* deletion displayed an enhanced blood lactate clearance following a lactate tolerance test as well as an increased production of glucose derived from lactate within the liver [146]. Moreover, these mice were protected against an otherwise lethal dose of lactate administered by injection [146]. In another study, it was found that mice with liver-specific *Phd2* KO (Alb-Cre) had a different metabolic response to exercise than mice with hepatocyte-specific *Hif1a* deletion [95].

Overall, PHD2 is a key regulatory enzyme for organismal energy metabolism and its inhibition may be protective against obesity and obesity-associated pathologies.

### Immune system

Conditional whole-body *Phd2* KO (Rosa26-CreERT2) in mice was protective against a lethal dose of LPS leading to an improved cardiac function, enhanced pericyte/EC coverage and increased survival [187]. In a model of bacterial pneumonia using *Streptococcus pneumonia*, myeloid-specific inactivation of *Phd2* (LysM-Cre) increased the inflammatory response resulting in increased lung injury [129]. This was caused by an enhanced neutrophil response, including an upregulated neutrophil functional capacity, motility and survival [129]. Mice with neutrophil-specific *Phd2* deletion (MRP8-Cre) also displayed an enhanced inflammatory response following treatment with LPS. Lack of *Phd2* augmented the neutrophil response through HIF-1 and increased the glycolytic flux [129]. In macrophages, *Phd2* deletion reduced their phagocytic and migratory capacity, which was HIF-1-dependent and mediated by a differential regulation of glycolytic enzymes [46]. Mice with *Phd2* haploinsufficiency showed no differential response to LPS-induced sepsis relative to control [69], which indicates that one functional *Phd2* gene locus is sufficient to maintain (certain) immune responses.

Myeloid-specific inactivation of *Phd2* (LysM-Cre) was also found to augment atherogenesis [166] and neointima formation [21]. Following a high-cholesterol diet, aortic root plaque size and macrophage apoptosis were increased in mice with *Phd2* deletion in myeloid cells [166].

Systemic knockdown (KD) of PHD2 using tetracycline-inducible shRNA as well as conditional global *Phd2* deletion (Rosa-ERTCre) resulted in leukocyte expansion and autoimmune features [183]. This phenotype was mediated through stabilisation of HIF-2α [183]. Regulatory T-cells (Tregs) from mice with *Phd2* KD/KO were dysfunctional, potentially underlying the observed increase in immune activity [183]. Treg-specific *Phd2* inactivation (Foxp3-Cre) led to a systemic inflammatory syndrome, including development of a rectal prolapse, shortening of the colon, splenomegaly and elevated IFN-γ expression [2]. This phenotype was mediated via HIF-2α [2]. In summary, PHD2 is an important regulator of Treg function, and its inactivation can lead to a severe dysregulation of the immune system.

Overall, PHD2 is an important regulator of immune cell function, which is at least in some cell types caused by the alteration of energy metabolism via HIF-1 stabilisation. Several investigations found an enhanced inflammatory response in both baseline or pathological conditions. Interestingly, this is overall not reflected in mouse models using PHIs [160]. Therefore, selective PHD2 deletion appears to have a different effect on the immune system than the inhibition of all three PHD isoforms (e.g. via PHIs).

### Lung

In lipopolysaccharide (LPS)-induced lung inflammation, murine-induced endothelial cell-specific *Phd2* deletion (Cdh5-CreER) was protective [38]. These animals displayed improved adherent junction integrity and endothelial barrier function, leading to reduced lung vascular permeability and inflammatory cell infiltration and preventing the formation of oedema [38]. This data suggests *Phd2* inhibition as a therapeutic strategy for acute lung inflammation. Haploinsufficiency of *Phd2* had no effect on pulmonary NEB number, but the NEB size was increased [116].

### Carotid body

*Phd2*^+*/−*^ mice demonstrated an increased ventilatory response to hypoxia with enlarged carotid bodies [9]. PHD2 was found to be the most important PHD isoform for the modulation of the hypoxia ventilatory response (HVR) [9]. Also, the induced global deletion of *Phd2* (Rosa26-CreERT2) led to an exaggerated HVR, which was mediated via HIF-2α [52]. A subsequent analysis of induced *Phd2* deletion in type I cells (tyrosine hydroxylase (TH) expressing cell lineage) of carotid bodies (tyrosine hydroxylase (TH), TH-IRES-CreER) found a multilineage expansion and features that resembled paragangliomas [40]. The observed changes of the carotid bodies were again dependent on HIF-2α [40]. Together, these analyses demonstrate an important regulation of carotid body hyperplasia and the HVR by PHD2 via HIF-2 with possible relevance for the development of paragangliomas.

### Nervous system

*Phd2*^+*/−*^ mice are protected from focal cerebral ischemia at 2 and 24 h following MCAO, displaying an enhanced restoration of cerebral blood flow (CBF) with improved functional outcomes, increased vascular density, less apoptotic cells and a reduced disruption of the blood–brain barrier [14]. Transient MCAO (acute I/R injury) in mice with constitutive neuron-specific *Phd2* KO (Ca^2+^/calmodulin-dependent protein kinase IIα promotor (CaMKIIα)-Cre) led to a decreased infarct size and cell death of hippocampal neurons compared to control [77]. HIF-1α and HIF-2α protein levels were increased in the forebrain combined with increased expression of Epo, VEGF and glycolytic enzymes [77]. Nonetheless, vessel density was not altered within forebrain subregions [77]. Assessment of the relevance of neuron-specific *Phd2* inactivation (CaMKIIα-Cre) on the recovery phase following MCAO also supports a protective effect. Mice with neuronal loss of *Phd2* showed a reduced infarct area, an increased vascular density along the infarct area, an improved sensory and motor function, an increase of VEGF expression and a reduction of pro-inflammatory cytokines [84]. Overall, *Phd2* inhibition is therefore a potential therapeutic strategy for ischemic brain injury.

Long-term potentiation (LTP) is a cellular mechanism considered to be of major relevance for learning and memory formation. Using the same constitutive neuron-specific *Phd2* KO mouse model as described above (CaMKIIα-Cre), it was shown that ablation of *Phd2* prevents mouse hippocampal LTP [23]. Thus, PHD2 inhibition may affect synaptic plasticity and therefore learning capabilities and memory formation. Interestingly, analysis of the cognitive function in the same murine neuron-specific *Phd2* deletion model (CaMKIIα-Cre) showed an enhanced spatial learning under both baseline conditions and following chronic brain hypoperfusion (permanent occlusion of the left common carotid artery) [45]. Increased cognitive function was associated with an increased number of neuronal precursor cells [45]. No change was observed in vascular density, expression of synaptic plasticity-related genes (in the hippocampus) or in the morphology of dendritic spines [45]. Therefore, whereas it has consistently been reported that *Phd2* deletion affects the activity of the hippocampus, the functional outcome of PHD2 inactivation is less clear. Of note, *Phd2*^+*/−*^ mice displayed no difference compared to wildtype mice in axonal regeneration following peripheral (sciatic) nerve injury, but showed a reduced latency in compound muscle action potentials, indicating an improvement of axon function [139].

Specific deletion of *Phd2* in NG2 glia cells and pericytes in the brain (NG2-Cre) had no effect on the brain vasculature [165]. Combinatorial PHD1-3 or VHL deletion in NG2 cells, however, led to an increase of capillary networks and proliferation of pericyte in several areas of the brain [165].

In summary, inactivation of *Phd2* affects cognitive functions, brain vessel density and HIF-mediated gene expression in neurons, which is protective in neuronal I/R injury.

### Skeletal muscle

In *Phd2*^+/*−*^ mice, ischemic injury by femoral artery ligation resulted in a comparably severe necrosis in the skeletal muscle as in control mice [4]. shRNA-mediated silencing of *Phd2* in tibial anterior and gastrocnemius muscles following right femoral artery ligation increased HIF-1α expression as well as the expression of VEGF and endothelial nitric oxide synthase (eNOS) [94]. Silencing of *Phd2* enhanced vessel and capillary density as well as macrophage infiltration into the ischemic muscle, indicating that *Phd2* inactivation supports muscle revascularisation [94]. The observed effects were likely mediated by enhanced HIF-1 activity [94]. Another investigation found that *Phd2*^+/−^ mice were protected against hindlimb ischemia-induced necrosis by preformed collateral arteries [153]. Arteriogenesis was improved due to an increase in tissue-resident M2-like macrophages and enhanced smooth muscle cell recruitment [153]. Acute and chronic *Phd2* haploinsufficiency in macrophages also led to arteriogenesis in the ischemic muscle, indicating that the protective effect of *Phd2* ablation was due to the resulting regulation of macrophages [153].

Induced keratinocyte-specific *Phd2* ablation (human keratin 14 promoter (KRT14)-CreERT) during femoral artery ligation improved distantly located vascular survival and arteriogenesis in ischemic hind limbs [151]. The protective effect could also be observed in type 1 and 2 diabetic mice and in mice with hepatocyte-specific *Phd2* KO (Alb-Cre) [151]. Local *Phd2* deletion selectively in keratinocytes of the hindlimb skin (by local administration of tamoxifen) was not protective against ischemic injury [151]. These results indicate that protection against ischemia by inactivation of *Phd2* works remotely and that it may therefore not be necessary to access the actual target tissue for treatment.

*Phd2* hypomorph mice showed increased HIF-1α and HIF-2α levels in skeletal muscles, increased capillary size without effects on capillary number and an upregulation of the expression of glycolytic genes [66]. After exercise, serum lactate levels were reduced faster [66]. Hind limb I/R injury led to reduced infarct size in *Phd2* hypomorphic mice, which was likely due to the increase in capillary size as well as the HIF-mediated regulation of energy metabolism [66].

Conditional whole-body *Phd2* KO mice demonstrated increased capillary density in skeletal muscle [113, 135] likely due to induction of VEGF [135]. Muscle fibres of KO animals transitioned towards slow type I fibres [135]. After mechanic muscle trauma, *Phd2* hypomorph mice presented with faster muscle tissue regenerative capabilities, including enhanced activation of myogenic factors, accelerated macrophage infiltration into injured tissue areas and upregulation of stem cell proliferation markers [134].

In summary, genetic PHD2 inhibition is protective in muscle ischemia due to enhanced arteriogenesis, which may be affected by increased VEGF secretion as well as by the induction of pro-angiogenic macrophages.

### Bone

Constitutive *Phd2* deletion in osteoblasts in mice (Col1a2‐iCre) led to premature death 12 to 14 weeks after birth [19]. These mice displayed a shorter stature with decreased bone mineral density, bone area and bone mineral content in tibias and femurs but not in vertebrae. Within the femoral trabecular bones, bone volume and total volume as well as bone volume fraction were reduced [19]. This phenotype was suggested to be caused by diminished bone formation [19]. There was no alteration in proximal tibial epiphyses in 5-week-old mice following Col1a2‐iCre-mediated *Phd2* deletion [17]. Conditional deletion of PHD2 in osteoprogenitors (OSX-Cre transgene) led to a less severe phenotype without the report of premature death and with the strongest changes observed with combinatorial deletions of PHD isoforms [173]. Conditional *Phd2* inactivation in haematopoietic cells, osteoblasts and Epo-producing cells (CD68-Cre) led to a strong decrease of bone density in the distal femur and in vertebrae [125]. Analyses of mice with *Phd2* deletion in osteoblasts (OSX-Cre) or osteoclasts (Vav-Cre) indicated that the observed bone malformations were not directly linked to an altered osteoblast or osteoclast activity caused by the inactivation of *Phd2* [125]*.* It was suggested that the bone malformations observed in mice with CD68-Cre-mediated *Phd2* deletion were caused by an Epo-mediated effect on osteoblast progenitors, because Epo levels were increased due to the deletion of *Phd2* [125]*.* Analyses in *Phd2*^+/−^ mice showed a decreased bone mineralisation and trabeculae bone mass [55].

Conditional deletion of *Phd2* in osteocytes (Dentin Matrix Protein 1 (Dmp1)-Cre) increased the bone mass by enhanced bone formation and reduced resorption [140]. In addition, these mice were protected against bone loss caused by a decline in oestrogen levels [140]. Moreover, *Phd2* ablation in osteocytes (Dmp1-Cre) increased fibroblast growth factor-23 (FGF23) levels (an important hormone regulating mineral ion handling) in the murine bone [112]. Deletion of *Phd2* in murine periosteum-derived cells improved bone regeneration following implantation of the KO cells due to enhanced cell viability, which was likely caused by an altered energy metabolism [141]. This phenotype was independent of angiogenesis [141].

Chondrocyte-specific *Phd2* conditional deletion (type 2 collagen-α1-Cre) led to enhanced trabecular bone mass of long bones by increased trabecular thickness and number with decreased trabecular separation [17, 20]. KO mice displayed increased bone formation rate in long bones, bone mineralisation and upregulated markers for chondrocyte hypertrophy [17, 20]. These results suggest that *Phd2* plays an important role in endochondral bone formation [17, 20]. Moreover, in the same mouse model, articular cartilage thickness was decreased, combined with increased chondrocyte differentiation [18].

In summary, PHD2 activity is of key importance for the regulation of bone volume and density as well as for articular cartilage thickness, likely including multiple mechanisms and cell types.

### Gastrointestinal tract

*Phd2*^+*/−*^ mice as well as mice with constitutive *Phd2* deletion in endothelial and haematopoietic cells (Tie2-Cre) did not show any difference compared to control mice in DSS-induced colitis [67, 154, 167]. Constitutive inactivation of *Phd2* in intestinal epithelial cells (Villin-Cre) did not lead to spontaneous intestinal inflammation and was also not protective in a DSS-induced colitis model or in colitis-associated colon cancer [177]. Deletion of *Phd2* in intestinal epithelial cells in mice was also not protective against radiation-induced gastrointestinal toxicity [157]. Mice with ablation of *Phd2* in regulatory T-cells (Foxp3-Cre) spontaneously developed systemic inflammation (see above) [2]. These mice also showed an increased sensitivity to toxoplasmosis and DSS-induced colitis, likely because of an inefficient control of the inflammatory response [2].

Analysis of anastomotic leakage in *Phd2*^+*/−*^ mice showed an improved healing of septic and ischemic colon anastomoses [144]. *Phd2* haploinsufficiency reduced anastomotic leakage, increased the bursting pressure and was protective against sepsis-related mortality [144]. This protective effect was achieved by the induction of M2 polarisation of macrophages, which reduced immune cell recruitment [144].

Overall, whilst *Phd2* is important for immune cell functions and thus for pro-inflammatory responses in vivo, *Phd2* is dispensable for intestinal development and intestinal epithelial homeostasis.

### Skin

The effect of *Phd2* deletion on wound healing was analysed using different mouse models. Constitutive *Phd2* KO in myeloid (LysM-Cre) or endothelial (Flk1-Cre) cells had no effect on wound closure in full-thickness excisional skin wounds (6-mm biopsy punches) [64]. In turn, specific constitutive *Phd2* deletion in keratinocytes (K14-Cre) decreased the time for wound closure and increased migration of the hyperproliferating epithelium as well as proliferation of keratinocytes in the *stratum basale* [64]. These effects were at least in part mediated by HIF-1 as well as by decreased transforming growth factor β signalling [64]. Mice with constitutive *Phd2* ablation in keratinocytes (K14-Cre) or induced *Phd2* deletion in fibroblasts (Col1α2-CreER) each showed an accelerated wound healing in 6-mm full-thickness excisional wounds [193]. Both epidermal and dermal *Phd2* KO were also protective in an ischemic pedicle flap model with more viable flaps being present in the *Phd2* KO mice in comparison to mice with wildtype *Phd2* alleles [193]. Injection of murine mesenchymal stromal cells transduced with shPHD2 into full-thickness excisional skin wounds also accelerated wound healing with enhanced cellularity and blood vessel density [70]. *Phd2* deletion in FoxD1-lineage mesodermal cells (FoxD1-Cre) led to truncal alopecia by disturbing hair follicle development [128]. Overall, *Phd2* inactivation in keratinocytes, fibroblasts and/or mesenchymal stem cells accelerates cutaneous wound healing. Thus, PHD2 is a promising potential therapeutic target for wound healing.

### Eye

Oxygen treatment of preterm infants can lead to retinopathy including loss of micro-vessels in the retina, which can result in blindness. In neonatal control mice, exposure to 75% O_2_ reduced retinal micro-vessels [31]. Induced global *Phd2* ablation (Rosa26-CreERT2) in mice from postnatal day 1 was protective against the effect of 75% O_2_ exposure on retinal micro-vessels with increased HIF-1α and HIF-2α protein levels compared to control mice [31]. This indicates that inhibition of PHD2 might be a therapeutic option for preterm infants with oxygen treatment to prevent retinopathy. Constitutive lack of *Phd2* in astrocytes (GFAP-Cre) resulted in an increased number of retinal astrocytes, impaired vascular pruning and increased HIF-2α protein levels in neonatal mice [30]. Therefore, the HIF pathway may play an important physiological role in retinal astrocytes for the appropriate development of the retinal vasculature.

### Cancer

Investigation of tumours derived from B16 melanoma, Panc02 pancreatic carcinoma or Lewis lung carcinoma (LLC) cells in *Phd2*^+*/−*^ mice showed no effect on tumour growth, tumour cell apoptosis or proliferation; however, the occurrence of metastasis was reduced [100]. In *Phd2*^+*/−*^ mice, tumour vessel endothelial lining and maturation were normalised (without effect on tumour vessel lumen size or density), preventing tumour cell intravasation, invasion and thus metastasis [100]. In mice with conditional haplodeficient *Phd2* inactivation in endothelial cells (Tie2-Cre), the main findings could be repeated, including reduced metastasis and tumour vessel normalisation [100]. Moreover, haplodeficient *Phd2* inactivation in endothelial cells in mice led to an improved chemotherapeutics delivery to tumours [81]. These results were repeated in mice with induced acute global heterozygous or homozygous deletion (Rosa26-CreERT2 transgene) of *Phd2* [81]. In addition, the induced global *Phd2* deletion led to an increased protection of healthy organs against detrimental side effects of chemotherapeutic agents in a HIF-dependent manner [81]. In a spontaneous metastatic mammary gland tumour model, *Phd2*^+*/−*^ mice reduced metastasis by reducing cancer-associated fibroblast (CAF) activation, production of extracellular matrix and CAF-mediated contraction [76]. This effect was dependent on tumour vessel normalisation as well as PHD2 inactivation in tumour cells and not in CAFs themselves [76].

Induction of primary hepatic tumours by diethylnitrosamine (DEN) treatment in *Phd2*^+*/−*^ mice resulted in enhanced hepatocarcinogenesis and increased development of cholangiocarcinoma with a larger number of metastasis, which was suggested to be caused by chronic HIF activation [49]. The beginning of neoplastic transformation was not altered by *Phd2* haploinsufficiency in the same hepatic tumour model, indicating that PHD2 activity is relevant during tumour nodule formation, but not for neoplastic transformation [10].

Analysing the relevance of PHD2 in immune cells for tumour development, *Phd2* was deleted in haematopoietic cells (CD68-Cre) and the mice were inoculated with LLC cells, leading to reduced tumour growth, decreased tumour cell apoptosis and enhanced proliferation [96]. In mice, *Phd2* deletion in myeloid cells (LysM-Cre), in all T-cell populations (CD4-Cre) or in B-cells (CD19-Cre) had no effect on tumours from LLC cells [96]. However, combined *Phd2* deletion in myeloid and T-cells (LysM/CD4-Cre) led to a decreased tumour growth [96]. This indicates that PHD2 activity in myeloid and T-cells supports tumour growth and that PHD2 inhibition may be a therapeutic option for lung cancer treatment.

Conditional melanocyte-specific deletion of *Phd2* (Tyr-CreER) did not lead to any pigmented lesions [92]. However, in mice with a melanocyte-specific deletion of *Phd2* combined with the expression of BRaf^V600E^, melanoma with a 100% penetrance and metastasis in lymph nodes were observed [92]. These results indicate that PHD2 can enhance melanomagenesis in the presence of BRaf^V600E^.

In mice with constitutive KO of *Phd2* in the medulla of the adrenal gland (TH-Cre), alterations in developmental adrenal morphologies were reported combined with a gene expression pattern mimicking pseudohypoxic pheochromocytoma [33]. The observed changes were shown to be HIF-2α-dependent. Interestingly, induced *Phd2* deletion in the adrenal medulla in adult mice did not lead to the aberrant gene expression pattern, demonstrating that the observed changes towards pseudohypoxic pheochromocytoma were likely occurring during adrenal gland development [33].

In a CAC model (AOM and DSS treatment), *Phd2*^+*/−*^ mice showed no difference in the induced colitis but CAC growth was enhanced together with the number of tumour-associated macrophages [67]. The observed regulation was due to an upregulation of the expression of epiregulin in macrophages, a ligand for EGFR, as well as an increased extracellular signal-regulated kinase 1/2 and signal transducer and activator of transcription 3 signalling [67].

In summary, these data indicate that inhibition of PHD2 activity alone is detrimental for some tumours, including lung carcinoma and prostate cancer, which is (at least in part) due to its effects on vessel formation, myeloid and T-cells. In melanocytes, PHD2 inhibition may be protective to a certain degree against the development of melanomas but is detrimental in the presence of BRaf^V600E^. Moreover, inactivation of PHD2 may support the growth and metastasis formation from hepatic tumour and cholangiocarcinoma as well as the growth of colon cancer. Interestingly, PHD2 activity appears to be necessary for the development of the adrenal gland, preventing a shift of the gene expression towards pseudohypoxic pheochromocytoma. Thus, PHD2 plays an important role in tumour growth and metastasis formation; however, its relevance and function depend on the cancer type.

## *Phd3 (Egln3****)*** deletion

The baseline phenotype summarises observations made in mice with *Phd3* deletion without the induction of a pathology. The subsequent chapters focus on phenotypes of mice with various *Phd3* deletions in disease models.

### Baseline phenotype

Mice with constitutive whole-body deletion of *Phd3* (*Phd3*^−/−^) are viable but with a small reduction in the offspring being observable after mating of heterozygous mice [8, 152]. At baseline conditions, there were no obvious abnormalities during development [152] or adulthood apparent [8, 149, 150] with physiological Epo and haematocrit levels as well as red blood cell counts [149], white blood cell counts (including normal neutrophil and macrophage numbers) [69, 169], angiogenesis [150] and no obvious alterations in the morphologies of lung and kidney [69]. Also, bone development and architecture were normal following the specific constitutive deletion of *Phd3* in osteoprogenitor cells (OSX-Cre) or chondrocytes (Col2α1-Cre) [173, 179]. In contrast, *Phd3*^−/−^ mice showed an increased trabecular spacing and a decreased trabecular number as well as a decreased fractional bone volume in long bones and vertebrae [55]. Mice with enzymatically inactive *Phd3* (R205K knock-in mutation) showed no obvious changes in development or fertility [62], similar to the *Phd3*^−/−^ mice.

Interestingly, *Phd3*^−/−^ mice display a neuronal phenotype [8]. The number of neurons was increased in the superior cervical ganglion (SCG), in the adrenal medulla and in the carotid body due to a decreased neuronal apoptosis [8]. Moreover, the function of the sympathoadrenal system was reduced with a decreased innervation of target tissues, secretory capacity of the adrenal medulla and reduced sympathoadrenal responses (e.g. of the iris, submandibular gland and pineal gland) combined with a decrease in systemic blood pressure [8]. The hypoxic ventilatory response was comparable to wildtype mice [9]. The phenotype was caused by the regulation of HIF-2α and was independent of HIF-1α [8]. Therefore, PHD3 plays a major role for the anatomic and functional integrity of the sympathoadrenal system [8]. *Phd3*^−/−^ mice also displayed hypertrophy and hyperplasia of NEBs with increased NEB size and NEB cells [116]. The NEB alterations were comparable to the findings in *Phd1* KO mice [116]. During aging, 1-year-old *Phd3*^−/−^ mice showed increased triglyceride and cholesterol levels, liver weight, adiposity and body weight, enhanced WAT inflammation and insulin resistance and hyperglycaemia [158].

Overall, *Phd3* deletion does not alter the majority of organ systems under baseline conditions. However, constitutive global *Phd3* ablation impacts on the development and function of the sympathoadrenal system with corresponding effects on systemic blood pressure, iris size modulation and excretion from glands. Moreover, during aging, PHD3 counteracts metabolic dysfunction.

### Cardiovascular system

To assess the relevance of PHD3 in ischemic heart injury, the response of *Phd3*^−*/*−^ mice to LAD ligation was investigated. In this model of myocardial infarction, *Phd3*^−*/*−^ mice showed improved cardiac function, increased capillary density, reduced cardiac fibrosis and increased HIF-1α DNA binding [114]. LAD ligation with subsequent release as model for I/R injury in induced whole-body *Phd3* KO mice (chicken β-actin-CreER) resulted in attenuated tissue damage [176]. Myocardial injury and apoptosis of cardiomyocytes were decreased [176]. In a rat model of type 2 diabetes (HFD plus streptozotocin injection), shRNA-mediated PHD3 knockdown (jugular vein injection) reduced cardiac dysfunction [174]. Thus, *Phd3* inactivation is protective in different models of cardiac injury, likely by decreasing cardiomyocyte apoptosis and by enhancing angiogenesis following injury.

Investigating further the relevance and function of PHD3 in the cardiovascular system, transgenic mice were generated with increased PHD3 expression. Cardiomyocyte-specific transgenic expression of PHD3 in mice (*cPhd3tg*) did not affect cardiac function or HIF activity at baseline conditions over an investigation period of 14 months [192]. Following LAD ligation, *cPhd3tg* hearts showed an increased infarct size linked to reduced HIF-1α and HIF-2α stabilisation [192]. In a mouse model of obstructive sleep apnoea, shPHD3 treatment had no effect, whereas lentiviral PHD3 overexpression reduced intermittent hypoxia-mediated cardiac perivascular collagen deposition and (partially) prevented cardiac dysfunction [188]. In a murine model of the effect of chronic intermittent hypoxia on cardiac pressure overload, lentiviral PHD3 overexpression improved the systolic function and alleviated cardiac remodelling [180]. These studies indicate that the effect of PHD3 overexpression may depend on the type of cardiac injury. The detrimental effect of PHD3 overexpression in cardiac ischemia further supports the finding that PHD3 deletion is protective.

In an atherosclerosis model, mice deficient for apolipoprotein E were fed HFD and either injected with lentivirus carrying shPHD3 or DNA for PHD3 overexpression [90]. PHD3 overexpression enhanced the area of aortic atherosclerotic lesions, the number of macrophages and smooth muscle cells and the number of apoptotic cells in atherosclerotic plaques [90]. Following a high-cholesterol diet in mice, *Phd3* and *Ldlr* double KO did not alter atherosclerotic plaque size or necrotic, macrophage, collagen or oxygen content [29].

In summary, PHD3 activity plays an important role in the response of cardiac tissue to injury and PHD3 may be a therapeutic target in myocardial infarction, diabetes-induced cardiac dysfunction and in cardiac injury caused by obstructive sleep apnoea. Its relevance in atherosclerosis is less clear.

### Haematopoietic system

There was no effect observed on erythropoiesis or haematopoiesis by constitutive global *Phd3* deletion [149], or *Phd3* ablation in hepatic [105, 163] or (renal) FoxD1 lineage cells [73]. Interestingly, in *Phd3* and *Ldlr* double KO mice, haematocrit was increased at baseline conditions whereas WBC counts were maintained [29].

### Liver

Following a partial hepatectomy, liver regeneration was not affected in *Phd3*^−*/*−^ mice [107]. In a model of chronic bile duct injury, *Phd3*^−*/*−^ mice showed no difference in biliary fibrosis [145]. Thus, PHD3 is not relevant for liver regeneration or (bile duct injury-mediated) hepatic fibrosis development or progression.

### Energy metabolism

Mice with liver-specific *Phd3* KO (tail vein injection of adenoviral Cre into *Phd3*^*fl/fl*^ mice) demonstrated lower fasting insulin and glucose levels as well as an increased glucose tolerance [156]. In addition, the hepatic expression of several gluconeogenic enzymes (*Pck1*, *G6pc*, *Ppargs1a*) was reduced as well as the expression of enzymes involved in lipid metabolism (*Srebf1c*, *Fas*). Mechanistically, the observed changes were linked to increased HIF-2α stabilisation and Irs2 expression in the liver [156]. To investigate the influence of hepatic *Phd3* depletion on diabetes, the mice were fed HFD. Both fasting blood glucose and fasting serum insulin improved in comparison to wildtype mice [156]. Liver-specific *Phd3* KO (Alb-Cre) decreased gluconeogenesis during fasting periods, which was mimicked in PHD3 His196Ala (inactive PHD3) knock-in mice [182]. Both mouse models were resistant against high-fat and high-sucrose diet-induced gluconeogenesis and hyperglycaemia with improved insulin and glucose tolerance tests [182]. The observed phenotypes were suggested to be caused by PHD3-mediated hydroxylation of CREB-regulated transcriptional coactivator (CRTC) 2 [182].

Mice with constitutive global *Phd3* deletion (CMV-Cre) displayed elevated fatty acid oxidation (FAO) in the skeletal muscle, especially after fasting [184]. Glycogen levels were maintained in the skeletal muscle, but they were reduced in the liver under normal feeding conditions. Under fasting conditions, O_2_ consumption and CO_2_ production were both enhanced whereas the respiratory exchange ratio (RER) was maintained [184]. In a strenuous exercise endurance challenge, both mice with global and skeletal muscle-specific (MCK-Cre) *Phd3* deletion displayed increased exercise capacity [184]. Mechanistically, PHD3 was reported to hydroxylate acetyl-CoA carboxylase (ACC) 2 (which converts acetyl-CoA into malonyl-CoA) and the hydroxylation of ACC2 reduced FAO [184]. Combinatorial deletion of *Phd3* and *Ldlr* in mice resulted in an increased body weight at baseline that was further significantly increased relative to control mice following high-cholesterol diet (HCD) [29]. After HCD, also enhanced triglyceride and plasma cholesterol levels were observed. In the liver, the expression of *Fas* and *Cyp7a1* was increased, which may contribute to the dyslipidaemia [29].

Pancreatic β-cell specific *Phd3* deletion (Ins-1-Cre) had no effect on glucose homeostasis under standard conditions [51, 109]. Nonetheless, β-cell mass was reduced and β-cell apoptosis was enhanced [51]. Following HFD, glucose homeostasis was impaired [109]. The metabolism of *Phd3*^−*/*−^ β-cells shifted from glycolysis to fatty acid oxidation, which was linked to reduced insulin secretion after prolonged HFD [109].

Overall, *Phd3* deletion protects against dietary-induced diabetes and according alterations in glucose homeostasis, which may be due to effects in the liver, on pancreatic β-cells and in the skeletal muscles. Moreover, the metabolic changes introduced by *Phd3* ablation support exercise endurance. FAO was increased in the skeletal muscle following *Phd3* KO, which may contribute to the protective effect against dietary-induced diabetes. These results principally support an approach to pharmacologically inhibit PHD3 as treatment for type 2 diabetes. However, one study reported that *Phd3* deletion in combination with *Ldlr* ablation leads to dietary-induced dyslipidaemia. Therefore, inactivation of PHD3 may also have detrimental effects in certain (metabolic) conditions.

### Immune system

The activity and function of neutrophils derived from *Phd3*^−*/*−^ mice were preserved in both normoxia and hypoxia; however, neutrophil apoptosis was increased leading to a reduced *Phd3*^−*/*−^ neutrophil survival during hypoxia [169]. In hypoxia, HIF transcriptional activity was not altered in *Phd3*^−*/*−^ neutrophils relative to control. The increase of apoptosis in hypoxia in *Phd3*^−*/*−^ neutrophils was linked to an increased expression of pro-apoptotic *Siva1* and a reduced hypoxia-mediated stimulation of the Siva1 target protein BCL-X_L_ [169]. In an LPS-induced acute lung injury model, neutrophil apoptosis was enhanced in *Phd3*^−*/*−^ mice, leading to a decreased total neutrophil count in the inflamed tissue [169]. In DSS-induced colitis, neutrophilic inflammation was also decreased [169]. These findings suggest that PHD3 plays a key role in neutrophil survival and neutrophil-driven inflammation.

The inflammatory response in *Phd3*^−*/*−^ mice was also assessed in models of abdominal sepsis. Following LPS- or bacterial-induced (caecal ligation and puncture model) sepsis, the survival of *Phd3*^−*/*−^ mice was reduced compared to WT, *Phd1*^−*/*−^ and *Phd2*^+*/−*^ mice [69]. In *Phd3*^−*/*−^ mice, plasma pro-inflammatory cytokine levels and macrophage recruitment to internal organs were increased during sepsis [69]. The decrease in survival during sepsis was linked to an increased activity of *Phd3*^−/−^ macrophages, which in turn was dependent on both HIF-1α and NF-κB activity [69]. *Phd3* deletion in macrophages altered their maturation and polarisation towards an M1 (pro-inflammatory) polarisation [69]. This increase in M1 polarisation was linked to an accelerated differentiation and was not observed in fully differentiated macrophages [147]. *Phd3*^−*/*−^ macrophages showed (similar to neutrophils) no change in HIF activity [147]. Interestingly, *Phd3* deletion, in contrast to its effect in neutrophils, decreased the apoptosis rate of macrophages [147]. Of note, in a model of hind limb ischemia in mice with myeloid-specific *Phd3* deletion (LysM-Cre), *Phd3*^−*/*−^ macrophages displayed an increase in M2 (anti-inflammatory) polarisation [6]. *Phd3* deletion in CD11c^hi^ cells (CD11c-Cre) did not affect dendritic cell maturation, metabolism or survival in basal or stimulated conditions [159]. In a mouse model of LLC cancer in mice with genetic *Phd3* inactivation (R205K mutation), macrophage M2 polarisation was prevented [62].

Mice with induced global *Phd3* ablation (chicken β-actin-CreER) were protected against ionizing radiation effects on the thymus with reduced apoptosis of thymus cells [175]. The protection was reported to be mediated by preventing the hydroxylation of HCLK2, which in turn reduced DNA damage-induced apoptosis [175].

In general, *Phd3* loss affects apoptosis in neutrophils and macrophages and therefore corresponding inflammatory responses. The effect of *Phd3* deletion on macrophage polarisation may depend on the inflammatory context as well as whether the deletion is present in all cells or is myeloid-specific. Further analyses will be necessary to clarify this.

### Lung

To assess the relevance of PHD3 for asthma pathogenesis, mice with constitutive selective *Phd3* KO or overexpression in CD11c^hi^ cells were generated (CD11c-Cre) [159]. In a model of allergic airway inflammation, no modulation of the induced asthma was found. Interestingly though, the CD11c-specific *Phd3* deletion prevented alveolar macrophages in competition with wildtype macrophages to optimally repopulate an empty alveolar niche [159].

### Nervous system

In a mouse model of cerebral ischemia using MCAO, constitutive global *Phd3* deletion aggravated regional cerebral blood flow, but did not change the functional outcome [14]. Thus, although PHD3 regulates neuronal apoptosis [8], it does not affect cerebral ischemic injury. Following peripheral (sciatic) nerve injury, *Phd3*^−*/*−^ mice demonstrated increased axonal regeneration and reduced cold hyperalgesia, indicating that pharmaceutical PHD3 inhibition may be a treatment option for peripheral nerve injury [139].

### Skeletal muscle

Several studies investigated the effect of different methods of *Phd3* deletion in a model of hind limb ischemia using femoral artery ligation. *Phd3*^−/−^ mice were not protected against ischemia-induced muscle cell death [4]. A separate study reported that shRNA-mediated knockdown of PHD3 enhanced vessel and capillary density as well as macrophage infiltration in the ischemic muscle [94]. These results were linked to increased HIF-1α stabilisation within the injury site [94]. Another investigation found that *Phd3*^−/−^ mice also display enhanced vessel and capillary density, improving motor function by enhanced recovery of perfusion as well as decreasing tissue fibrosis [126]. Mechanistically, HIF-1α was increased combined with enhanced VEGF and Bcl-2 expression [126]. Murine myeloid-specific *Phd3* deletion (LysM-Cre) had no effect on angiogenesis or the recovery of reperfusion, but led to a decreased infiltration of macrophages and to a reduced fibrosis in the ischemic muscle [6].

Using viral transduction of sgRNA targeting PHD3 in mice with skeletal muscle-specific Cas9 expression (MCK-Cre-Cas9), the induced PHD3 deletion increased the area of muscle fibres and overall muscle weight [82]. In mice with denervated muscle atrophy, sgRNA-mediated *Phd3* KO mitigated the loss of muscle weight and the reduction of muscle fibres [82]. It was suggested that *Phd3* deletion enhances the activity of the major transcriptional regulator of the cellular response to pro-inflammatory signals, NF-κB [82]. It has further been reported that muscle-derived stem/progenitor cells (MDSPC) from *Phd3*^−*/*−^ mice display an increased myogenic potential [137].

Overall, the majority of reports support a protective effect of genetic *Phd3* inactivation in hind limb ischemia, leading to increased vascularisation and decreased fibrosis. In addition, *Phd3* deletion may also be counteracting muscle atrophy following denervation. Therefore, pharmacologic inhibition of PHD3 appears to be a promising approach for the treatment of various muscle pathologies.

### Gastrointestinal tract

Investigating the relevance of PHDs for the development and progression of inflammatory bowel diseases, it was demonstrated that global homozygous *Phd3* deletion in mice does not affect susceptibility or development of DSS-induced colitis [154]. *Phd3* KO in haematopoietic and endothelial cells (Tie2-Cre) did also not influence DSS-induced colitis in mice [167]. In a model for colitis-associated colorectal cancer (AOM and DSS treatment), *Phd3*^−*/*−^ mice showed no difference in disease activity or CAC growth [67]. In contrast, another study found that mice with *Phd3* deletion in intestinal epithelial cells (Villin-Cre) spontaneously develop colitis and demonstrate an increased disease activity in DSS-induced colitis [16]. This phenotype was suggested to be caused by the PHD3-dependent regulation of the tight junction protein occludin [16]. The same group reported in a later study again that intestinal epithelial cell-specific *Phd3* ablation was detrimental in DSS-induced colitis, but that surprisingly knock-in of catalytically inactive PHD3 (H196A) had no effect on disease activity, body weight development or shortening of the colon [181]. It was further shown that PHD3 regulates goblet cell generation in the murine intestine, which was suggested to occur independent of PHD3 enzymatic activity [181].

In a model of radiation-induced gastrointestinal toxicity, it was shown that ablation of *Phd3* alone in the murine intestinal epithelium (Villin-Cre) had no effect on the progression or outcome of the toxicity [157]. In a model of intestinal anastomoses and anastomotic leakage, constitutive global *Phd3* KO strongly enhanced gross structural anastomotic defects in mice [144].

In summary, there is strong evidence that PHD3 is dispensable in DSS-induced colitis (and CAC), whereas one group reported a functional relevance for PHD3 in colitis. In accordance with the majority of reports about the function of PHD3 in intestinal inflammation or injury, PHD3 is also not relevant for radiation-induced gastrointestinal toxicity. Interestingly, selective genetic inactivation of *Phd3* is detrimental in intestinal anastomoses. Overall, PHD3 is likely dispensable in pathologies caused by intestinal epithelial barrier dysfunction.

### Cancer

Inactivation of *Phd3* by R205K knock-in mutation in mice reduced LLC cancer growth through regulation of Erk3, which is involved in the EGFR signalling pathway [62]. Inactivation of *Phd3* prevented macrophage efferocytosis, migration and M2 polarisation [62]. Analysing the relevance of PHD3 in CAC, *Phd3*^−*/*−^ mice displayed no difference in CAC growth [67].

Overall, to the best of our knowledge, the relevance of host *Phd3* for cancer development and progression is not very well studied using KO mice. The existing studies indicate that host PHD3 activity supports LLC cancer growth whereas it is dispensable for CAC.

## Conclusions

The cellular oxygen sensors PHD1-3 are regulatory proteins within the HIF pathway, regulating HIF-α protein stability. Cellular studies have indicated distinct roles for each of the PHDs for the regulation of HIF-1α and HIF-2α. Importantly, knockdown and knockout in rodents of either of the genes encoding *Phd1*, *Phd2* or *Phd3* have demonstrated an independent function and relevance of each isoform on the organismal level (Tables 1 and 2). In baseline conditions, PHD1 is not essential during development and has a key function in the regulation of oxidative metabolism in the skeletal muscle via HIF-2α (Table 1). PHD2 is the most relevant PHD isoform for physiology, as constitutive and induced global *Phd2* deletion is lethal both during development and adulthood, whereas the ablation of PHD1 or PHD3 is well tolerated (Table 1). PHD2 is also the most relevant regulatory enzyme of HIF-α in vivo, as highlighted in mice lacking PHD2 by, e.g. increased erythropoiesis, angiogenesis and developmental heart defects. PHD3 is essential for the appropriate development of the sympathoadrenal system (Table 1).

**Table 1:** Relevance of PHD1-3 in mammals under normal housing conditions. Summary of phenotypes in rodents with the deletion or silencing of the respective *Phd* gene. If not indicated otherwise, phenotypes observed in mice with homozygous gene deletion are described. NEB, neuroepithelial bodies; WAT, white adipose tissue; shPDH2, short hairpin (sh)RNA targeting PHD2; Epo, erythropoietin; RBC, red blood cells; BAT, brown adipose tissue; IFN-γ, interferon γ; SCG, superior cervical ganglion

	Organ (system), physiological process	Gene inactivation/silencing	Phenotype	Reference
PHD1	Energy metabolism	Constitutive whole-body (*Phd1*^*−/−*^)	↓ Oxygen consumption in skeletal muscle, ↓ whole-body oxygen consumption, ↓ exercise endurance	[4]
			↑ WAT mass, ↓ liver mass, ↓ food intake, ↓ insulin sensitivity	[162]^#^
	Energy metabolism during aging (1-year-old)	*Phd1*^*−/−*^	↓ Serum cholesterol levels, ↓ body weight	[158]
	Lung	*Phd1*^*−/−*^	↑ Size and frequency of NEBs	[116, 117]
	Pancreas	β-cells	↓ β-cell mass and elevated β-cell apoptosis	[51]
PHD2	Embryogenesis	Constitutive whole-body *Phd2*^*−/−*^, induced somatic homozygous, injection of lentivirus carrying shPHD2 into one-cell murine zygotes	Lethal due to placental and heart defects	[104, 115, 152]
	Aging	Hypomorph	↓ liver diseases, ↓ inflammation, ↓ myocardial infarction, ↑ diastolic function (1-year old mice) and developed, ↓ cardiomyocyte hypertrophy	[78, 127]
	Cardiovascular system	Induced somatic	Premature death due to dilated cardiomyopathy and venous congestion	[104]
			↑ Angiogenesis, angiectasia	[150]
	Haematopoietic system	Induced somatic; Epo-producing cells, neurons and astrocytes; renal cortical interstitial cells; haematopoietic progenitor cells; FoxD1 lineage cells; injection of adenovirus encoding a Cre enzyme into the tail vein of Phd2^flox/flox^ mice	↑ Plasma Epo, ↑ RBC count, ↑ haemoglobin, ↑ haematocrit	[5, 41, 73, 86, 104, 105, 113, 121, 149, 152]
		Induced somatic	Erythrocytosis	[86]
		Induced somatic	↑ White blood cell number (in peripheral blood), ↑ haematopoietic stem cells and progenitors (in spleen, liver, bone marrow)	[149]
		Haematopoietic precursor cells	↑ Self-renewal of multipotent haematopoietic progenitors	[136]
	Kidney	Endothelial cells	↑ Serum creatinine, ↑ glomerular arteriolar remodelling, ↑ interstitial fibrosis	[170]
	Liver	Induced somatic; hepatocytes	Hepatic steatosis	[105]
		Endothelial cells	Hepatic steatosis, liver fibrosis	[190]
	Energy metabolism	Induced somatic	↑ Effect of endurance training, ↑ running time	[113]
		Induced somatic, hepatocytes, hypomorph	↓ Lactate levels after exercise, ↑ blood lactate clearance	[66, 146]
		hypomorph	↓ Adipocyte size, ↓ WAT weight, ↓ WAT inflammation, ↑ insulin sensitivity, ↑ glucose tolerance, ↓ de novo lipid synthesis, ↓ serum cholesterol levels	[122]
		Adipocytes	↑ Adiposity and adipose vascularisation, ↓ circulating fatty acid levels	[102]
		BAT specific by viral *in-situ* injection containing targeted sgRNA against *Phd2*	↓ Reduced BAT thermogenesis in cold temperatures	[83]
		Pancreatic β-cells	↑ Plasma insulin	[51]
	Immune system	Induced somatic	↓ Phagocytosis and migration of macrophages	[46]
		Myeloid cells	↑ Atherogenesis	[166]
			↑ Neointima formation	[21]
		Induced somatic; tetracycline-induced﻿ systemic knockdown via shPHD2	↑ Leukocyte expansion, dysfunctional regulatory T-cells	[183]
		Regulatory T (Treg) cells	↑ Rectal prolapse, shortening of the colon, splenomegaly, elevated IFN-γ expression	[2]
	Carotid body	constitutive whole-body heterozygous (*Phd2*^+*/−*^); induced somatic	↑ Ventilatory response to hypoxia	[9, 52]
		*Phd2*^+*/−*^	↑ Size of carotid bodies	[9]
		Tyrosine hydroxylase expressing cell lineage	Multilineage expansion of type I cells, development of features that resemble paragangliomas	[40]
	Nervous system	Neurons	↓ Hippocampal long-term potentiation	[23]
			↑ Spatial learning	[45]
	Skeletal muscle	Hypomorph	↑ Capillary size	[66]
		Induced somatic	↑ Capillary density	[113, 135]
			↑ Slow type I fibres	[135]
	Bone	*Phd2*^+*/−*^	↓ Bone mineralisation, ↓ trabeculae bone mass	[55]
		Osteoblasts	Premature death with shorter stature, decreased bone mineral density, bone area and bone mineral content in tibias and femurs; reduction of femoral trabecular bone volume	[19]
		Osteocytes	↑ Bone mass via ↑ bone formation and ↓ bone resorption	[140]
		Chondrocytes	↑ Trabecular long bone mass via ↑ trabecular thickness and number with ↓ trabecular separation; ↑ long bone formation, ↑ bone mineralisation	[17, 20]
		Chondrocytes	↓ Articular cartilage thickness, ↑ chondrocyte differentiation	[18]
	Skin	FoxD1 lineage cells	Disturbed hair follicle development, truncal alopecia	[128]
	Eye	Astrocytes	↑ Retinal astrocyte number, defective vascular pruning	[30]
PHD3	Embryogenesis	Constitutive whole-body (*Phd3*^*−/−*^)	Small reduction in the number of offspring from heterozygous mating	[8, 152]
	Energy metabolism during aging (1-year-old)	*Phd3*^*−/−*^	↑ Serum triglyceride levels, ↑ serum cholesterol levels, ↑ liver weight, ↑ adiposity, ↑ body weight, ↑ WAT inflammation, hyperglycemia, insulin resistance	[158]
	Bone	*Phd3*^*−/−*^	↑ Trabecular spacing, ↓ trabecular number	[55]
	Nervous system	*Phd3*^*−/−*^	↑ Neuron numbers in SCG, adrenal medulla and carotid body, ↓ neuronal apoptosis, ↓ function of sympathoadrenal system	[8]
	Lung	*Phd3*^*−/−*^	Hypertrophy and hyperplasia of NEBs	[116]

^#^Other groups did not report comparable findings despite performing similar experiments (see text for details)

**Table 2:** Relevance of PHD1-3 in mammals in diseases and organ injuries. Summary of phenotypes in rodents with the deletion or silencing of the respective *Phd* gene in the indicated injury or disease models. If not indicated otherwise, phenotypes observed in mice with homozygous gene deletion are described. Only systemic *Phd* gene deletions or methods of gene silencing with systemic effects have been considered in the table, as these are most relevant for considerations of possible effects for the application of pharmacologic PHD inhibitors. The phenotypes of rodents with conditional *Phd1-3* deletion is described in the text. I/R, ischemia/reperfusion; *Ldlr*, low-density lipoprotein receptor; MCAO, middle cerebral artery occlusion; lncRNA, long non-coding RNA; MIAT, myocardial infraction associated transcript; WAT, white adipose tissue; BBB, blood–brain barrier; CAF, cancer-associated fibroblast; shPHD3, short hairpin (sh)RNA targeting PHD3; LPS, lipopolysaccharide; LAD, left anterior descending artery; DSS, dextran sulphate sodium

	Organ (system), process	Gene inactivation/silencing	Disease model	Phenotype	Reference
PHD1	Cardiovascular system	Constitutive whole-body (*Phd1*^*−/−*^)	Myocardial I/R injury (Langendorff’s perfusion)	↓ Infarct size, ↓ cardiomyocyte apoptosis	[1]
			Hypercholesterolaemia and artherosclerosis (*Ldlr* deficiency)	↓ Atherosclerotic plaque size, ↓ plasma cholesterol levels	[97]
	Liver	*Phd1*^*−/−*^	I/R injury	↓ Organ damage, ↓ hepatocyte swelling and apoptosis, ↓ vascularisation, ↓ karyolysis	[130]
			Chronic bile duct injury	↓ Liver fibrosis, ↓ inflammatory leukocytes	[145]
			80% hepatectomy	↑ Liver weight recovery, ↑ hepatocyte proliferation	[107]
			Low-fat diet (LFD)	Hepatic steatosis, ↑ hepatic cholesterol and triglyceride content	[162]
		Simultaneous tail vein injection of shPHD1 and shKeap1	Liver fibrosis (carbon tetrachloride treatment)	↓ Fibrosis	[91]
	Energy metabolism	*Phd1*^*−/−*^	LFD	↑ Body weight gain, ↓ insulin sensitivity	[162]
			High-fat diet (HFD)	↑ Body weight gain, ↑ insulin sensitivity, ↓ blood glucose and cholesterol	[162]
	Nervous system	*Phd1*^*−/−*^	Cerebral ischemia (MCAO)	↓ Infarct size	[120]
			Peripheral (sciatic) nerve injury	↓ Cold hyperalgesia, ↑ axonal regeneration	[139]
		Intravenous injection of shPHD1 combined with lncRNA MIAT overexpression	Cerebral I/R injury (MCAO)	↓ Injury, abrogation of detrimental effects of MIAT overexpression	[85]
	Skeletal muscle	*Phd1*^*−/−*^	Hind limb ischemia (femoral artery ligation)	↓ Injury (almost no necrosis or apoptosis)	[4]
				↑ Motor function, improver recovery of perfusion, ↑ arteriolar and capillary density, ↓ fibrosis	[126]
	Gastrointestinal tract	*Phd1*^*−/−*^	DSS-induced colitis	↓ Disease activity, ↑ colonic epithelial cell density, ↑ epithelial barrier function, ↓ epithelial cell apoptosis, ↓ weight loss	[67, 154]
	Skin	*Phd1*^*−/−*^	Acute inflammation (12-O-tetradecanoylphorbol-13-acetate treatment)	↓ Inflammatory response, ↑ apoptosis	[164]
	Cancer	*Phd1*^*−/−*^	Triple-negative breast cancer	↑ Survival	[148]
			Colon-associated colorectal cancer (CAC)	↓ CAC growth	[67]
PHD2	Cardiovascular system	Hypomorph	Myocardial I/R injury (Langendorff’s perfusion)	↓ Infarct size, ↑ recovery of coronary flow, ↑ mechanical function	[56]
		Hypomorph	Myocardial I/R injury (LAD ligation)	↓ Infarct size, improved preservation of the systolic function (left ventricle), ↑ survival	[68]
		Intraperitoneal injection of siPHD2	Myocardial I/R injury (Langendorff’s perfusion, LAD ligation)	↓ Acute myocardial injury, ↓ infarct size, ↓ infiltration of polymorphonuclear leukocytes	[110, 111]
		Induced global shPHD2 expression	Myocardial I/R injury (LAD ligation)	↓ Infarct size, ↑ left ventricular ejection fraction, fractional area shortening (effects varied dependent on time of shRNA expression relative to onset of injury)	[61]
		Hypomorph	HFD combined with *Ldlr* deficiency	↓ Atherosclerotic plaque areas, ↓ WAT macrophages	[123]
		Induced somatic	HFD-induced cardiac dysfunction	↑ Cardiac function	[186]
	Liver	Hypomorph	Alcoholic fatty liver disease (AFLD)	Protected against liver damage and steatosis, ↓ adiposity, improved lipoprotein profile, ↓ WAT inflammation	[79]
	Energy metabolism	Hypomorph	HFD	↓ Adipocyte size, ↓ WAT weight, ↓ WAT inflammation, ↑ insulin sensitivity, ↑ glucose tolerance, ↓ de novo lipid synthesis, ↓ serum cholesterol levels	[122]
		Hypomorph	HFD combined with *Ldlr* deficiency	↓ Body weight gain, ↓ WAT and liver weight, ↓ insulin resistance, ↓ adipocyte size, ↓ cholesterol levels, ↓ macrophage number in WAT	[123]
		Induced somatic	HFD	↓ Body weight gain, ↑ glucose tolerance	[186]
	Immune system	Induced somatic	LPS-induced sepsis	Protection against lethal dose, ↑ cardiac function	[187]
	Nervous system	Constitutive heterozygous whole-body (*Phd2*^+*/−*^*)*	Cerebral ischemia (MCAO)	Protected against focal cerebral ischemia, ↑ cerebral blood flow restoration, improved functional outcome, ↑ vascular density, ↓ apoptotic cells, ↓ BBB disruption	[14]
	Skeletal muscle	*Phd2*^+*/−*^	Hind limb ischemia (femoral artery ligation)	Protected against necrosis, ↑ arteriogenesis, tissue-resident M2-like macrophages, ↑ smooth muscle cell recruitment	[153]
		Hypomorph		↓ Infarct and capillary size	[66]
		Hypomorph	Mechanic muscle trauma	↑ Muscle regeneration, accelerated macrophage infiltration	[134]
	Gastrointestinal tract	*Phd2*^+*/−*^	Anastomotic leakage	↑ Healing of septic and ischemic colon anastomoses, ↑ M2 macrophage polarisation, ↑ bursting pressure, protective against sepsis-related mortality	[144]
	Eye	Induced somatic	Retinopathy by exposure to 75% O_2_	Protective against loss of retinal micro-vessels	[31]
	Cancer	*Phd2*^+*/−*^	B16 melanoma, Panc02 pancreatic carcinoma, Lewis lung carcinoma (LLC)	↓ Metastasis, ↑ tumour vessel endothelial lining and maturation, ↓ tumour intravasation and invasion	[100]
		*Phd2*^+*/−*^, induced somatic heterozygous and homozygous	B16 melanoma, LLC	↑ Chemotherapeutics delivery to tumours	[81]
		*Phd2*^+*/−*^	Metastatic mammary gland tumours with features of human ductal breast cancer (Polyoma virus middle T antigen overexpression)	↓ Metastasis, ↓ CAF activation, ↓ extracellular matrix production, ↓ CAF-mediated contraction, ↑ tumour vessel maturation	[76]
		*Phd2*^+*/−*^	Hepatic tumours (induced by diethylnitrosamine treatment)	↑ Hepatocarcinogenesis, ↑ cholangiocarcinoma, ↑ metastasis	[49]
		*Phd2*^+*/−*^	CAC	↑ Tumour growth, ↑ tumour associated macrophages	[67]
PHD3	Cardiovascular system	Constitutive whole-body (*Phd3*^*−/−*^)	Myocardial ischemia (LAD ligation)	↑ Cardiac function, ↑ capillary density, ↓ cardiac fibrosis	[114]
		Induced somatic	Myocardial I/R (LAD ligation)	↓ Myocardial injury, ↓ cardiomyocyte apoptosis	[176]
		jugular vein injection of shPHD3	rat model of type 2 diabetes (HFD plus streptozotocin injection)	reduced cardiac dysfunction	[174]
	Energy metabolism	*Phd3*^*−/−*^	*Ldlr* deficiency	↑ Body weight	[184]
			*Ldlr* deficiency and high-cholesterol diet	↑ Body weight gain, ↑ triglyceride and plasma cholesterol levels	[184]
	Immune system	*Phd3*^*−/−*^	Hypoxia	↑ Neutrophil apoptosis, ↓ neutrophil survival	[169]
			LPS-induced acute lung injury	↑ Neutrophil apoptosis, ↓ neutrophil count	[169]
			DSS-induced colitis	↓ Neutrophilic inflammation	[169]
			Abdominal sepsis (LPS- and bacterial-induced)	↓ Survival, ↑ plasma pro-inflammatory cytokine levels, ↑ macrophage recruitment to internal organs, ↑ macrophage M1 polarisation	[69]
		Induced somatic	Ionising radiation of thymus	Protective, ↓ apoptosis of thymus cells	[175]
	Nervous system	*Phd3*^*−/−*^	Cerebral ischemia (MCAO)	↓ Regional cerebral blood flow, no change in functional outcome	[14]
			Peripheral (sciatic) nerve injury	↑ Axonal regeneration, ↓ cold hyperalgesia	[139]
	Skeletal muscle	*Phd3*^*−/−*^	Hind limb ischemia (femoral artery ligation)	↑ Vessel and capillary density, ↑ recovery of perfusion, ↑ motor function, ↓ tissue fibrosis	[126]
	Gastrointestinal tract	*Phd3*^*−/−*^	Intestinal anastomoses and anastomotic leakage	↑ Gross structural anastomotic defects	[144]

The deletion of either of the PHD isoforms can lead to the stabilisation of HIF-1α or HIF-2α in vivo. However, the observed regulation of HIF-α levels appears to be cell-type and/or organ-specific in mice with *Phd1* and *Phd3* deletion and may additionally be disease/injury-specific. Some phenotypes in mice with *Phd1* or *Phd3* KO were linked to regulations independent of HIF, suggesting a functional relevance of the enzymatic function of the PHDs outside the HIF pathway. Nonetheless, additional investigations will be necessary to clarify this.

Interestingly, inactivation of *Phd1* or *Phd3* within mice was protective (or had no effect) against tumour growth and metastasis formation in the assessed cancers. Even *Phd2* ablation was protective in some cancer types, whereas it may be detrimental in hepatic and colon cancer. HIF activity can support tumour growth [106, 172]; therefore, a major concern for the clinical use of PHD inhibitors is a putative supportive effect for cancer development or progression. However, the results from PHD KO mice suggest that it may be possible to target these enzymes without detrimental effects regarding tumour growth. Of note, treatment with (non-selective) hydroxylase inhibitors is protective in various tumour models [43].

Currently, no PHD isoform selective pharmacologic inhibitors are available and the clinical use of the existing PHIs for hypoxia-associated diseases other than renal anaemia is currently prevented by the enhancement of Epo expression. Epo appears to be the most sensitive gene in response to HIF activation; therefore, PHIs can be used at relatively low doses for the treatment of renal anaemia. However, the sensitivity of the Epo expression to PHD inhibition is a disadvantage for the treatment of other hypoxia-associated diseases with these PHIs, as a strong increase in erythropoiesis is in many diseases not necessarily desirable. Some diseases may still be treatable, if a targeted local release is possible without systemic exposure to the PHI. Otherwise, single and combinatorial pharmacologic targeting especially of PHD1, PHD3 and also FIH will need to be considered and investigated further, as systemic pharmacologic inhibition of these oxygen sensors can be protective in different diseases without eliciting an Epo response.

Overall, gene deletions in mice of *Phd1*, *Phd2* or *Phd3* have highlighted the physiological relevance especially of PHD2 and the great potential of these three enzymes as pharmacologic targets in many different hypoxia-associated diseases. PHD isoform-selective inhibitors would thus offer a unique possibility for the treatment of various hypoxia-associated diseases and their development would spark an exciting research area for their potential use as indicated by the numerous isoform-selective deletion models.

## Data Availability

Not applicable.
